# The Effect of Yoga on the Lipid Profile: A Systematic Review and Meta-Analysis of Randomized Clinical Trials

**DOI:** 10.3389/fnut.2022.942702

**Published:** 2022-07-14

**Authors:** Dorsa Ghazvineh, Mojtaba Daneshvar, Vahid Basirat, Elnaz Daneshzad

**Affiliations:** ^1^Department of Physical Education, Islamic Azad University of Karaj, Karaj, Iran; ^2^Department of Community Nutrition, School of Nutritional Sciences and Dietetics, Tehran University of Medical Sciences, Tehran, Iran; ^3^Department of Gastroenterology, School of Medicine, Isfahan University of Medical Sciences and Health Services, Isfahan, Iran; ^4^Non-communicable Diseases Research Center, Alborz University of Medical Sciences, Karaj, Iran

**Keywords:** yoga, exercise, lipid profile, systematic review, meta-analysis

## Abstract

**Objectives:**

Yoga is a mind-body stress-relieving exercise that increases mental and physical health, which may have a role in the improvement of metabolic disorders. The present study has reviewed the effect of yoga on lipid profiles as a systematic review and meta-analysis.

**Methods:**

We evaluated the available randomized controlled trials on the effects of yoga-based programs, and lipid profiles by searching PubMed/Medline, Scopus, Web of Science, and the Cochrane central register of control trials up to January 2022. Both fixed and random effect analyses were used to find the relationships. Subgroup analysis was performed based on the continent, duration of the included studies, gender, and health condition of participants to discover the sources of heterogeneity.

**Result:**

Fifty-three studies were included in the current systematic review and meta-analysis with a total sample size of 13,191. There was a striking association between yoga and total cholesterol (−10.31 mg/dl; 95% CI: −14.16, −6.45; *I*^2^ = 82.5%, *P* < 0.001), low-density lipoprotein cholesterol (−8.64 mg/dl; 95% CI: −12.03, −5.25; *I*^2^ = 75.0%, *P* < 0.001), high-density lipoprotein cholesterol (1.98 mg/dl; 95% CI: 0.81, 3.14; *I*^2^ = 91.6%, *P* < 0.001), triglycerides (−13.50 mg/dl; 95% CI: −20.09, −6.92; *I*^2^ = 90.7%, *P* < 0.001) and very low-density lipoprotein (−3.94 mg/dl; 95%CI: −6.31, −1.56; *I*^2^ = 72.2%, *P* < 0.001).

**Conclusion:**

It seems yoga interventions had a substantial effect on lipid profiles, however, more qualified trials or cohort studies are needed to conclude exactly.

## Introduction

Modernization has brought increased comforts and limited mobility in our lives at the cost of an increased prevalence of hypertension, diabetes mellitus, dyslipidemia, and obesity, which are predecessors of major cardiovascular diseases (CVD) (1). Evidence suggests most of these diseases were rare before the present century and their prevalence has increased over the past 50 years (1). Also, metabolic syndrome (MetS) has been described as a pandemic, with a rapidly increasing prevalence worldwide (2). While dyslipidemia is a contributing risk factor for various macrovascular complications, MetS and CVD, which is characterized by high levels of triglyceride (TG ≥ 150 mg/dl), high low-density lipoprotein (LDL-C ≥ 130 mg/dl), low high-density lipoprotein (HDL-C < 40 mg/dl for men; < 50 mg/dl for women), and high levels of total cholesterol (TC ≥ 200 mg/dl) (3). Both prevention and control of coronary heart disease and its associated diseases are essential and can be achieved by modifying the lipid profile (4). Given the increasing prevalence and the associated premature mortality, disability, and health and social-economic costs of chronic diseases which are related to serum lipid levels, its management is of importance to public health (5). Not only healthy dietary patterns and a healthy lifestyle are effective on serum lipid levels but also physical activity and management of mental stress play an integral role in this area.

One of the best exercises that could help with physical and mental health is yoga. It was born in India thousands of years ago and has gradually expanded throughout the world. Yoga is a range of effective alternatives to traditional aerobic and strength training programs, which require little space, no equipment, and have limited side effects. Components of yoga that are commonly applied for health benefits are asanas (physical postures), pranayama (regulated breathing), meditation, relaxation, and various physical postures. Yoga calms and relaxes the mind, strengthens, and tunes the body, and brings them into harmony with one another (1). Deep relaxation, a unique part of a yoga program, relaxes the sympathetic nervous system and helps with physiological stress reduction. Physiological stress itself is related to metabolic disease (6). It seems yoga by improving physiological stress, will help cure cardiometabolic risk factors such as blood pressure, lipid, and glucose levels, as well as body weight (6). Even more, a study reported a better lipid profile in long and medium-term meditators when compared to non-meditators (7). Despite these claims, there is some evidence that shows that high-intensity yoga has no significant effects on cardiovascular outcomes or any of the blood parameters (8). Therefore, this review aims to systematically assess the effects of yoga on blood lipid levels, including through randomized clinical trials (RCTs).

## Methods

This study was conducted and reported according to PRISMA guidelines (preferred reporting items for systematic reviews and meta-analyses) (9).

### Search Strategy

A comprehensive literature search was conducted using PubMed, Scopus, Web of Science, Google Scholar, and Cochrane databases. The query was based on a combination of text words and terms from the Medical Subject Headings (MeSH): [Yoga(tiab) OR Yoga(MeSH) OR yogic OR yog* OR “yogasana” OR “surya namaskar” OR “vinyasa” OR “Thai yoga” OR “asana” OR “hatha” OR “pranayama” OR Pranayam* OR “dhyana” OR “Laughter therapy”(tiab) OR “Laughter therapy”(MeSH) OR “mind-body” OR “mind-body therapies”(tiab) OR “Mind-Body Therapies”(MeSH) OR “Mindfulness-based interventions” OR “Mindful exercise” OR “Exercise therapy”(tiab) OR “Traditional Chinese exercise” OR “Mindfulness interventions”(tiab) OR “complementary therapies”(tiab) OR meditation OR mindfulness OR “mindfulness-based stress reduction”] AND [“lipid profile” OR “serum lipids” OR “blood lipids” OR lipoproteins OR lipoprotein OR lipids OR Hypercholesterolemia(MeSH) OR Hyperlipidemias(MeSH) OR Hypercholesterolemia OR Hyperlipidemias OR “serum lipid markers” OR hypertriglyceridemia OR dyslipid* OR HDL OR HDL-C OR “Lipoproteins, HDL”(MeSH) OR “Cholesterol, HDL”(MeSH) OR “HDL Cholesterol” OR “high density lipoprotein” OR “high-density LDL-C OR “Lipoproteins, LDL”(MeSH) OR “Cholesterol, LDL”(MeSH) OR “LDL Cholesterol” OR “Low density lipoprotein” OR “low-density lipoprotein” OR “LDL-cholesterol” OR “Low Density Lipoprotein Cholesterol” OR TAG OR TG OR Triglyceride OR Triglycerides(MeSH) OR triacylglycer* OR TC OR Cholesterol OR “total cholesterol” OR Cholesterol(MeSH)].

All articles published before January 2022 were searched and examined by two authors to determine whether they were eligible for the present systematic review and meta-analysis. First, the titles and abstracts of the studies were reviewed to find articles related to our research question. If it was not certain whether or not the study met the inclusion criteria, the full text was reviewed to clarify this issue. Bibliographies of eligible studies and relevant reviews were also checked to reduce the possibility that a publication had been overlooked. All of the above steps were performed independently by two authors. Any discrepancies, from study selection to data extraction, were resolved in consultation with the lead author.

### Inclusion and Exclusion Criteria

RCTs that investigated the effect of yoga on lipid profiles were included in this review. Eligibility criteria for inclusion in this study were defined as follows: if the study design was RCT (parallel/crossover), conducted in adult subjects (> 18 years), reporting mean and standard deviation (SD) outcomes at baseline and the end of the study or mean changes between the intervention and control groups. Only articles published in English were included in the present study. In addition, the following exclusion criteria were defined, namely, observational studies, non-interventional studies, studies without a placebo group, studies in children, lactating or pregnant women, animal studies, gray literature (books, letters, commentaries, and conferences), as well as dissertations and reviews were excluded.

### Data Extraction

Two reviewers (DG and MD) independently scanned the articles for titles and abstracts. Any discrepancies between these two authors were clarified by a third researcher (ED) as the principal investigator. In this study, the effect of yoga was considered as an intervention. Moreover, the mean and SD of HDL-C, LDL-C, very-low-density lipoprotein (VLDL), TG, and TC were the outcomes. Data were extracted from each included study, including the first author of the study, date of publication, type of study, population, number of participants in intervention and control groups, gender of participants, age of participants at baseline, study location, duration of intervention, and body mass index (BMI) as well as mean and SDs of lipid criteria before and after the intervention.

### Risk of Bias

Based on the Cochrane guideline (10), we evaluated the quality of the studies by the following criteria: random sequence generation, allocation concealment, blinding, incomplete outcome data, selective outcome reporting, and other possible sources of bias. According to this guideline, studies were categorized as low risk or high risk of bias or unclear regarding each domain (Supplementary Table 1).

### Statistical Analysis

The means and corresponding SDs of all variables of all included studies in both intervention and control groups were used to calculate the weighted mean difference (WMD) as effect size in the meta-analysis. For studies that did not report mean changes, we computed this variable using mining pre-and post-intervention data. Also, the SD of the mean difference was calculated by the following formula: SD = [(SD_baseline_^2^ + SD_final_^2^) – (2 × R × SD_baseline_^2^ × SD_final_^2^)], (R-value = 0.5) (11). In cases where SD was not reported, we calculated SD using SE and sample size (SD = SE × √sample size). The reported rate of lipid profile in all studies was converted into the usual unit (mg/dl). The analysis was performed using the fixed-effect model. Also, the random effect model was used for variables with high heterogeneity. Subgroup analyses by continent, duration, gender, and condition were performed using Cochran’s *Q* test and the *I*^2^ statistic to assess the possible sources of heterogeneity. In addition, publication bias was assessed using funnel plots and Egger’s regression test. A sensitivity analysis was performed to determine the extent to which summary estimates might be related to a particular study or group of studies and also meta-regression test was used to determine the effect of age confounded. Data analyses were performed using Stata Software, version 14. *P*-values were reported as statistically significant at the < 0.05 level.

## Results

### Literature Review

**FIGURE 1:**
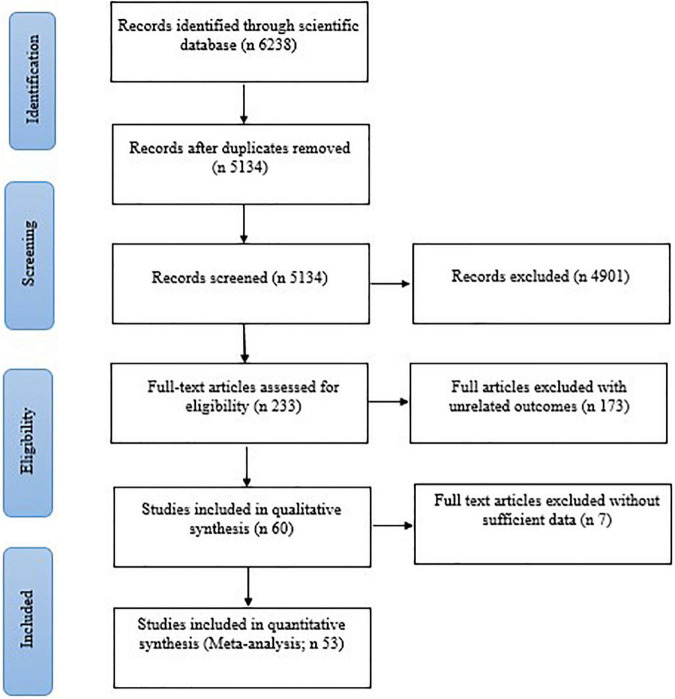
Flow diagram showing the selection of RCT studies for the current systematic review and meta-analysis of the effect of yoga exercise on lipid profile.

After screening the titles and abstracts of 6,238 articles, about 233 studies were adopted to be assessed for full-text. After excluding unrelated and review studies, 53 studies were kept and included in this systematic review (Figure 1). All studies employed a parallel design. Characteristics of the included studies, which were published from 1991 to 2021 are illustrated in Table 1. The sample size of these studies varied from 8 to 8,116 (3) and overall 13,191 participants, divided into 6,700 individuals in the control group and 6,517 in the intervention group. The age range was between 18 and 70 years old. Of the 53 included studies, 36 were conducted in India (3, 7, 12–44), but two effect sizes were extracted from Rani et al. (13), four effect sizes from Murthy et al. (25), and two effect sizes from P. A et al. (27), as well as six in the United States (6, 45–49), and three in China (2, 5, 50). Seven studies were conducted on women (2, 19, 37, 49, 51–53), six on men (6, 16, 18, 22, 31, 34), and the remaining studies were conducted on both genders (3, 5, 7, 12–15, 17–21, 23, 25–30, 32, 33, 35, 37–48, 50, 54–59). Included 37 studies had assessed BMI (2, 5–7, 12, 13, 15–19, 21, 22, 24, 27–31, 33, 34, 36–38, 40–43, 46–49, 52, 53, 56–58), 48 studies had measured TC (2, 3, 6, 7, 12–43, 45–47, 49, 52–59),4 6 studies had determined LDL-C (2, 3, 6, 7, 12–42, 45–47, 49, 52–58), 51 studies had measured HDL-C (2, 3, 5–7, 12–21, 23–54, 56, 58, 59), 53 studies had determined TG (2, 3, 5–7, 12–59), and 19 studies had assessed VLDL-C (14, 16, 18, 19, 24, 26, 28, 29, 31, 32, 34–40, 42, 55). These outcomes were reported as mean ± SD, also a meta-analysis as reported below was conducted. RCTs were performed on 11 studies with healthy situations (2, 21, 34, 39, 41, 45, 47, 49, 52, 56, 58), and 19 studies with type 2 diabetes mellitus (3, 6, 7, 12, 13, 16–20, 22–24, 27, 33, 35, 37, 38, 55), 10 studies with MetS (5, 26, 36, 42–44, 46, 48, 50, 51), 5 studies with heart diseases (MI, CAD, CVD) (29–31, 33, 54), 4 studies with hypertension (14, 25, 53, 57), 3 studies with chronic kidney disease (CKD) (15, 28, 59), 1 study with human immunodeficiency virus (HIV) (54) and 1 with obesity (40).

**TABLE 1:** Characteristics of included studies in the current systematic review and meta-analysis on lipid.

References	Country	Number of participants	Age (years)	BMI	Gender	Outcome	Main condition	Duration (week)
			(Mean±SD)					
Blumenthal et al. (45)	USA	57	67±4.9	–	both	TC,HDL,LDL,TG	Healthy	56
		Int:30						
		Con:27						
Agte (43)	India	65	Int:	Int:	both	HDL,TG, TC	metabolic syndrome	16
		Int:35	54.6±11.8	25.4±3.7				
		Con:30	Con:	Con:				
			55.7±5.8	25.6±4.3				
Yurtkuran et al. (59)	Turkey	37	Int:	–	both	TC,HDL,TG	Hemodialysis	6
		Int:19	38±14.2					
		Con:18	Con:					
			41±9.97					
Cohen et al. (46)	USA	24	52±9	36±6	both	TC,HDL,LDL,TG	metabolic syndrome	10
		Int:12	Int:	Int:				
		Con:12	52±9	37±6				
			Con:	Con:				
			52±8	35±6				
Gordon et al. (55)*	Cuba	154	Int:64	–	both	TG,HDL,	T2DM	24
		Int:77	Con: 63.6			TC,VLDL,LDL		
		Cont:77						
Singh et al. (37)*	India	60	45±16.88	Int:	both	TG,HDL,TC,	T2DM	6
		Int:30		26.12±1.54		VLDL,LDL		
		Con:30		Con:				
				25.83±1.77				
Cade et al. (54)	Australia	50	Int:	–	both	TC,HDL,LDL,TG	HIV+CVD	20
		Int:29	45±6					
		Con:21	Con:					
			45±10					
Pal et al. (29)	India	154	Int:	Int:	both	TG,HDL,TC,	coronary artery disease	24
		Int:80	58.9±9.4	24.46±4.2		VLDL,LDL		
		Con:74	Con:	Con:				
			58.6±10.5	25.19±4.5				
Yang et al. (6)	USA	23	51.7±4.9	29.79±5.24	males	TC,HDL,LDL,TG	T2DM	12
		Int:12		Int:				
		Con:23		28.2±3.7				
				Con:				
				31.5±6.2				
P (27)*	India	38	47.5±4.76	Int:	Both	TC,HDL,LDL,TG	T2DM	12
		Int:16		27.86±1.54				
		Con:22		Con:				
				25.84±1.76				
P (27)*	India	28	47.5±4.76	Int:	both	TC,HDL,LDL,TG	T2DM	12
		Int:6		26.86±1.46				
		Con:22		Con:				
				25.84±1.77				
Mizuno and Monteiro (57)	Brazil	33	Int:	Int:	both	TC,LDL,TG	hypertension	16
		Int:17	67±7	27.4±4.4				
		Con:16	Con:	Con:				
			62±12	26.4±5.3				
Vaishali (17)	India	65	Int:	Int:	both	TC,HDL,LDL,TG	T2DM	12
		Int:35	65.8±3.2	27.12±2.13				
		Con:30	Con:	Con:				
			64.4±3.8	28.14±1.38				
Subramanian et al. (39)	India	40	20.5±1.87	–	both	TG,HDL,TC,	Healthy	6
		Int:21				VLDL,LDL		
		Con:19						
Hunter et al. (47)	USA	33	Int:	Int:	both	TC,HDL,LDL,TG	Healthy	12
		Int:14	49±5	29±4				
		Con:19	Con:	Con:				
			49±6	31±7				
Nagarathna et al. (26)*	India	173	Int:	–	both	TG,HDL,TC,	metabolic syndrome	48
		Int:88	53.46±8.86			VLDL,LDL		
		Con:85	Con:					
			51.38±8.39					
Lee et al. (52)	Korea	16	54.5± 2.75	Int:	Female	TC,HDL,LDL,TG	Healthy	16
		Int:8	Int:	25.13±1.63				
		Con:8	54.75±2.76	Con:				
			Con:	25.19±1.71				
			54.25±2.91					
Gordon et al. (15)*	India	66	Int:	Int:	both	TC,HDL,LDL,TG	ESRD	16
		Int:33	38.95±2.84	25.55±2.21				
		Con:33	Con:	Con:				
			44.59±2.57	25.74±0.5				
Rani et al. (13)*	India	70	Int:	Int:	both	TC,HDL,LDL,TG	T2DM	12
		Int:33	64±4	25.3±3.4				
		Con:37	Con:	Con:				
			62±3.4	25±4.1				
								
Rani. et al. (13)*	India	47	Int:	Int:	both	TC,HDL,LDL,TG	T2DM	12
		Int:26	64±4	25.3±3.4				
		Con:27	Con:	Con:				
			62±3.4	25±4.1				
								
Rani et al. (13)*	India	26	Int:	Int:	both	TC,HDL,LDL,TG	T2DM	12
		Int:14	64±4	25.3±3.4				
		Con:12	Con:	Con:				
			62±3.4	25±4.1				
								
Bindra et al. (20)	India	100	50±9.09	–	both	TC,HDL,LDL,TG	T2DM	12
		Int:50						
		Con:50						
Kim et al. (51)	Korea	37	Int:	–	Female	TG, HDL	metabolic syndrome	24
		Int:17	48.2±7.21					
		Con:20	Con:					
			50.3±8.3					
Shantakumari et al. (7)	India	100	Int:	Int:	both	TC,HDL,LDL,TG	T2DM	12
		Int:50	45.51±7.98	22.9±2.15				
		Con:50	Con:	Con:				
			44.46±10.98	23.2±2.14				
Raghuram et al. (31)	India	165	Int:	Int:	males	TG,HDL,TC,	coronary artery disease	48
		Int:89	53.34±6.42	26.76±3.24		VLDL,LDL		
		Con:76	Con:	Con:				
			52.6±6.85	25.22±3.15				
Kanaya et al. (48)	USA	135	Int:	Int:	both	TG, HDL	metabolic syndrome	48
		Int:72	55±7	36±7.3				
		Con:63	Con:	Con:				
			54±7	32.5±5.9				
Telles et al. (40)	India	44	36.4±11.2	Int:	both	TG,HDL,TC,	Obesity	2
		Int:22	Int:	38.23±6.81		VLDL,LDL		
		Con:22	36±10.3	Con:				
			Con:	35.65±6.35				
			36.8±12.1					
Wolff et al. (53)*	Sweden	43	Int:	Int:	Females	TC,HDL,LDL,TG	hypertension	12
		Int:21	66.2±7.7	29.7±7				
		Con:22	Con:	Con:				
			60.8±11	28.8±4				
Wolff et al. (53)*	Sweden	42	Int:	Int:	Females	TC,HDL,LDL,TG	hypertension	12
		Int:20	64±10.3	29.7±7				
		Con:22	Con:	Con:				
			60.8±11	28.8±4				
Thiyagarajan et al. (41)	India	100	Int:	Int:	both	TC,HDL,LDL,TG	Healthy	12
		Int:51	44.08±9.42	25.74±3.52				
		Con:49	Con:	Con:				
			42.47±9	25.71±3.21				
Kumpatla et al. (19)	India	241	Int:	Int:	both	TG,HDL,TC,	T2DM	12
		Int:131	41±8.7	27.2±4.1		VLDL,LDL		
		Con:110	Con:	Con:				
			44.2±7.4	27±4.5				
lau et al. (5)	China	154	Int:	Int:	both	TG,HDL	metabolic syndrome	12
		Int:79	52.44±7.15	24.44±3.48				
		Con:75	Con:	Con:				
			51.52±7.78	25.9±3.9				
Siu et al. (50)	China	182	56±9.1	–	both	TG,HDL	metabolic syndrome	24
		Int:84	Int:					
		Con:98	56.3±8.8					
			Con: 55.7±9.4					
Ruby et al. (49)	USA	18	43.2±4.6	26.7±4.5	Females	TC,HDL,LDL,TG	Healthy	12
		Int:8	Int:	Int:				
		Con:10	44.8±4.5	26.3±4.4				
			Con:	Con:				
			41.6±10.4	25.4±4.3				
Chen et al. (2)	China	30	53±2	Int:	Females	TC,HDL,LDL,TG	Healthy	12
		Int:15		20.55±2.01				
		Con:15		Con:				
				20.68±2.01				
Shete et al. (34)	India	36	41.5±5.2	21.1±3.6	males	TG,HDL,TC,	Healthy	12
		Int:18				VLDL,LDL		
		Con:18						
Hewett et al. (56)	Australia	63	37.2±10.8	30.5±6.2	both	TC,HDL,LDL,TG	Healthy	12
		Int:29	Int:	Int:				
		Con:34	38.2±10.1	29.9±6.2				
			Con:	Con:				
			36.3±11.4	30.9±6.3				
Manna (21)	India	60	21±15.27	Int:	both	TC,HDL,LDL,TG	Healthy	12
		Int:30		22.5±1.2				
		Con:30		Con:				
				20.5±1.7				
Mondal et al. (24)	India	20	64.4±4.79	24.28±2.36	Females	TG,HDL,TC,	T2DM	12
		Int:10	Int:	Int:		VLDL,LDL		
		Con:10	64.7±4.03	24.26±3.4				
			Con:	Con:				
			64.4±4.79	24.28±2.36				
Dutta et al. (28)	India	60	Int:	Int:	both	TG,HDL,TC,	CKD	12<
		Int:30	55.1±11.6	22.7±3		VLDL,LDL		
		Con:30	Con:	Con:				
			55.6±11.2	23.3±2.5				
Murthy et al. (25)*	India	35	45±11.97	–	both	TC,HDL,LDL,TG	hypertensive diabetic	48
		Int:15						
		Con:20						
Murthy et al. (25)*	India	98	45±11.97	–	both	TC,HDL,LDL,TG	hypertensive non-diabetic	48
		Int:69						
		Con:59						
McDermott et al. (22)	India	38	Int:	Int:	males	LDL,TC,TG	T2DM	8
		Int:20	47±9.7	28.4±5.3				
		Con:18	Con:	Con:				
			47.2±9.1	26.9±3				
Murthy et al. (25)*	India	11	45±11.97	–	both	TC,HDL,LDL,TG	prehypertensive diabetic	48
		Int:7						
		Con:4						
Murthy et al. (25)_*_	India	62	45±11.97	–	both	TC,HDL,LDL,TG	prehypertensive non-diabetic	48
		Int:32						
		Con:30						
Singh et al. (36)*	India	26	Int:	Int:	Females	TG,HDL,TC,	metabolic syndrome	12
		Int:14	51.77±8.73	27.99±3.49		VLDL,LDL		
		Con:12	Con:	Con:				
			53.8±8.30	28.16±3.06				
Tillin et al. (58)	UK	80	Int:	Int:	both	TC,HDL,LDL,TG	Healthy	12
		Int:40	57.4±1.65	27.6±1.09				
		Con:40	Con:	Con:				
			56.9±1.55	27.2±6.93				
Yadav et al. (44)	India	260	Int:	–	both	TG,HDL	metabolic syndrome	12
		Int:130	37.7±6.3					
		Con:130	Con:					
			37.6±6.4					
Viswanathan et al. (42)	India	300	Int:	Int:	both	TG,HDL,TC,	metabolic syndrome	12
		Int:150	52.8±7	28.1±4.5		VLDL,LDL		
		Con:150	Con:	Con:				
			50.8±8.3	28.1±4.8				
Arumugam et al. (12)	India	146		26.69±4.58	both	TC,HDL,LDL,TG	T2DM	24
		Int:73	55.61±10.9	Int:				
		Con:73		27.18±34.75				
				Con:				
				26.16±43.23				
Sharma et al. (33)*	India	64	Int:	–	both	TC,HDL,LDL,TG	Coronary	12
		Int:32	53.15±11.59				Artery Disease	
		Con:32	Con:					
			51.51±8.15					
Sharma (32)*	India	104	50±11.67	Int:	both	TG,HDL,TC,	T2DM	24<
		Int:52		28.14±3		VLDL,LDL		
		Con:52		Con:				
				28.15±3				
Prasad et al. (30)	India	200	Int:	Int:	both	TC,HDL,LDL,TG	Myocardial	24
		Int:100	59.83±11.41	28.36±3.5			infarction	
		Con:100	Con:	Con:				
			60±11.53	29.46±4.83				
Shetty (35)	India	60	47.5±10.53	–	both	TG,HDL,TC,	T2DM	<2
		Int:30				VLDL,LDL		
		Con:30						
Biswas (14)	India	40	Int:	–	both	TG,HDL,TC,	hypertension	12
		Int:20	45.75±8.63			VLDL,LDL		
		Con:20	Con:					
			45.75±8.63					
Gupta et al. (16)	India	78	50.6±8.5	27.9±4.7	males	TG,HDL,TC,	T2DM	16
		Int:34	Int:	Int:		VLDL,LDL		
		Con:40	51.1±8.6	28.8±5.2				
			Con:	Con:				
			50.2±8.6	27.1±4.1				
Nagarathna et	India	8116	Int:	–	both	TC,HDL,LDL,TG	T2DM	12
al. (3)*		Int:3933	48.7±10.64					
		Con:4183	Con:					
			48.41±10.22					
Sivapuram et al. (38)	India	81	Int:	Int:	both	TG,HDL,TC,	T2DM	12
		Int:50	58.86±24.73	26.51±4.18		VLDL,LDL		
		Con:31	Con:	Con:				
			53.31±7.71	28.51±5.11				
Kaur et al. (18)	India	182	Int:	Int:	males	TG,HDL,TC,	T2DM	12
		Int:92	47.77±9.59	28.59±5.75		VLDL,LDL		
		Con:92	Con:	Con:				
			49.24±10.53	28.53±5.01				
Misra et al. (23)	India	321	53.3±10.7	–	both	TC,HDL,LDL,TG	T2DM	12
		Int:164	Int:					
		Con:157	52.8±10.1					
			Con:					
			54.2±11.2					

*indicates consecutive studies by the same authors that come from just one article but with different situations, such as differences in number or condition.

*Int: intervention group; Con: control group; BMI: body mass index; TC: total cholesterol; TG: triglyceride; LDL: low-density lipoprotein; HDL: high density lipoprotein; VLDL: very low-density lipoprotein; CKD: chronic kidney disease; ESRD: End-Stage Renal Disease; HIV: human immunodeficiency virus; CVD: Cardiovascular disease; T2DM: type 2 diabetes mellitus.*

### Meta-Analysis

#### Total Cholesterol

**FIGURE 2:**
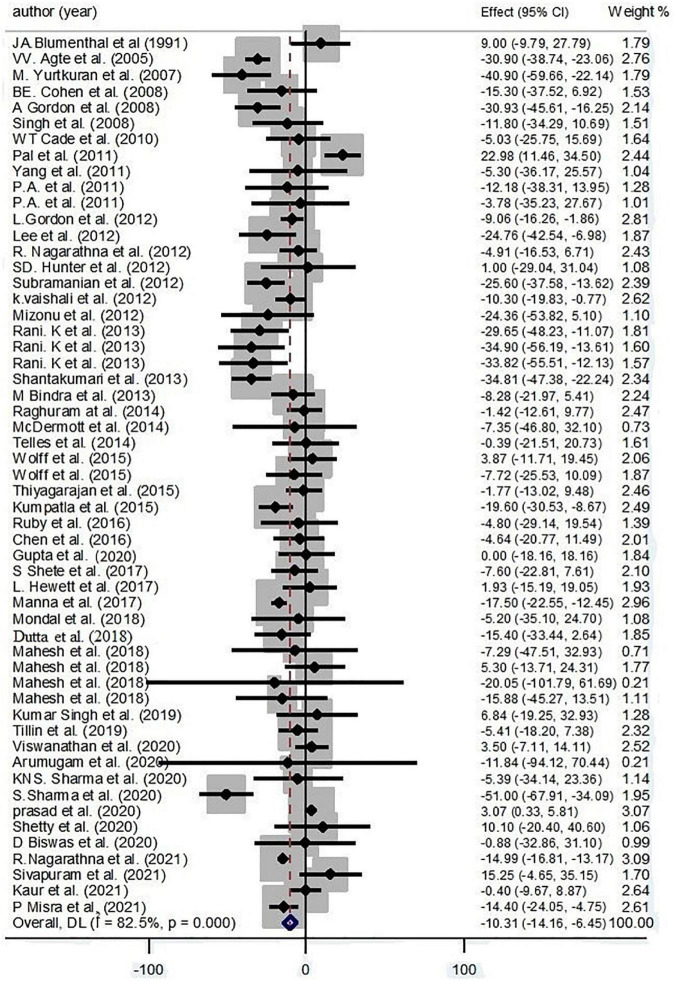
Forest plot for the association between yoga exercise and total cholesterol (Random-effect model).

Findings from 55 effect sizes have shown an inverse effect of yoga on TC with high heterogeneity by pooling amounts (−10.31 mg/dl; 95% CI: −14.16, −6.45; *P* < 0.001; *I*^2^ = 82.5%, *P*_heterogeneity_ < 0.001) (Figure 2).

Because of high heterogeneity, groups were subdivided according to different factors. Based on subgroup analysis, continent, condition, and gender were considered as the main sources of heterogeneity for TC. Results revealed that yoga significantly decreased TC levels among American (−13.6 mg/dl; 95% CI: −21.39, −4.77; *P* = 0.002; *I*^2^ = 54.3%; *P*_heterogeneity_ = 0.41) and Asian (−10.51 mg/dl; 95% CI: −11.74, −9.28; *P* < 0.001; *I*^2^ = 85.5%; *P*_heterogeneity_ < 0.001) yoga workers. In addition, yoga had a negative effect on TC levels in healthy practitioners (−11.61 mg/dl; 95% CI: −16.13, −9.09; *P* < 0.001; *I*^2^ = 60.1%; *P*_heterogeneity_ = 0.005) and patients with MetS (−14.68 mg/dl; 95% CI: −19.94, −9.41; *P* < 0.001; *I*^2^ = 87.9%; *P*_heterogeneity_ < 0.001) and CKD (−13.43 mg/dl; 95% CI: −19.73, −7.13; *P* < 0.001; *I*^2^ = 79.4%; *P*_heterogeneity_ = 0.008) (Supplementary Table 2).

Based on the result of meta-regression, no effect was found for age on the effect size (regression coefficient = −0.05; 95%CI: −0.65, 0.53; *P* = 0.839) (Supplementary Figure 1).

According to the result of Egger’s test and funnel plot of 55 studies, no evidence of publication bias was found (*P* = 0.918) (Supplementary Figure 2). Moreover, the sensitivity analysis of included trials did not affect the final pooled amounts.

#### Low-Density Lipoprotein Cholesterol

**FIGURE 3:**
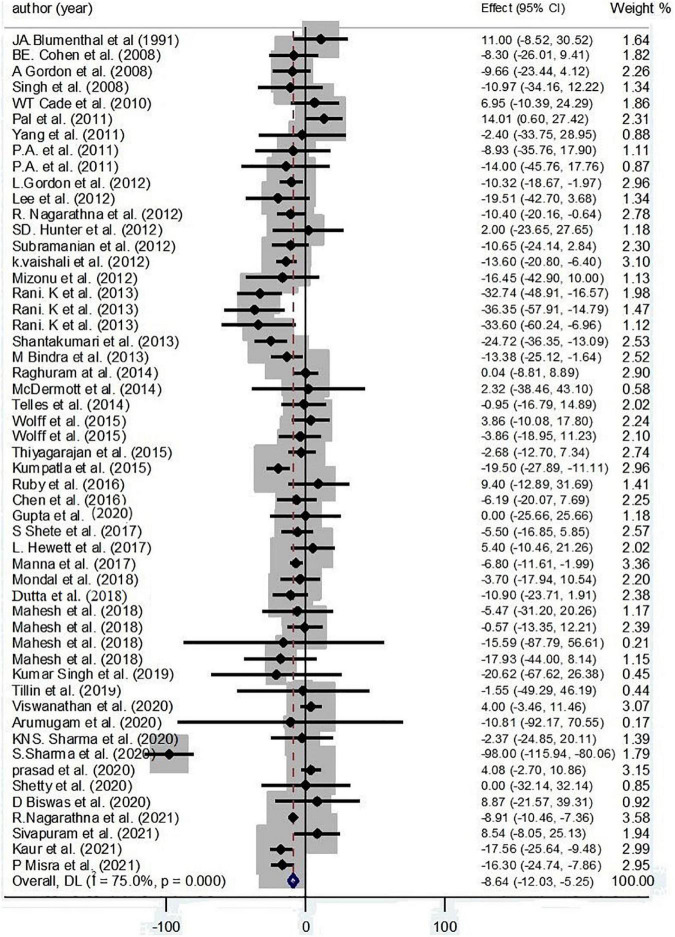
Forest plot for the association between yoga exercise and LDL-C (Random-effect model).

An inverse effect of yoga on LDL-C has been detected by collecting 53 effect sizes (−8.64 mg/dl; 95% CI: −12.03, −5.25; *P* < 0.001; *I*^2^ = 75.0%, *P*_heterogeneity_ < 0.001) (Figure 3).

Based on subgroup analysis, continent, condition, duration, and gender were considered as the main sources of heterogeneity. The subgroup analysis based on continent has cleared an inverse effect of yoga on LDL-C among Asian participants (−8.92 mg/dl; 95% CI: −10.11, −7.72; *P* < 0.001; *I*^2^ = 79.1%, *P*_heterogeneity_ < 0.001). Moreover, pooling effect sizes have revealed that more than 12 weeks of yoga interventions decreased LDL-C levels (−8.57 mg/dl; 95% CI: −9.75, −7.38; *P* < 0.001; *I*^2^ = 78.2%, *P*_heterogeneity_ < 0.001). Also, an inverse effect of yoga on LDL-C levels was found among healthy practitioners (−4.98 mg/dl; 95% CI: −8.43, −1.53; *P* = 0.005; *I*^2^ = 60.1%, *P*_heterogeneity_ = 0.005) and patients with CKD (−10.49 mg/dl; 95% CI: −17.49, −3.50; *P* = 0.003; *I*^2^ = 0.0%, *P*_heterogeneity_ = 0.941) and type 2 diabetes (−10.76 mg/dl; 95% CI: −12.14, −9.38; *P* < 0.001; *I*^2^ = 84.4%, *P*_heterogeneity_ < 0.001) (Supplementary Table 3).

Based on the result of meta-regression, no effect was found for age on the effect sizes (regression coefficient = −0.15; 95%CI: −0.63, 0.31; *P* = 0.508) (Supplementary Figure 3).

According to the result of Egger’s test and funnel plot of 53 studies, no evidence of publication bias was found (*P* = 0.981) (Supplementary Figure 4). Moreover, the sensitivity analysis of included trials did not affect the final pooled amounts.

#### High-Density Lipoprotein Cholesterol

**FIGURE 4:**
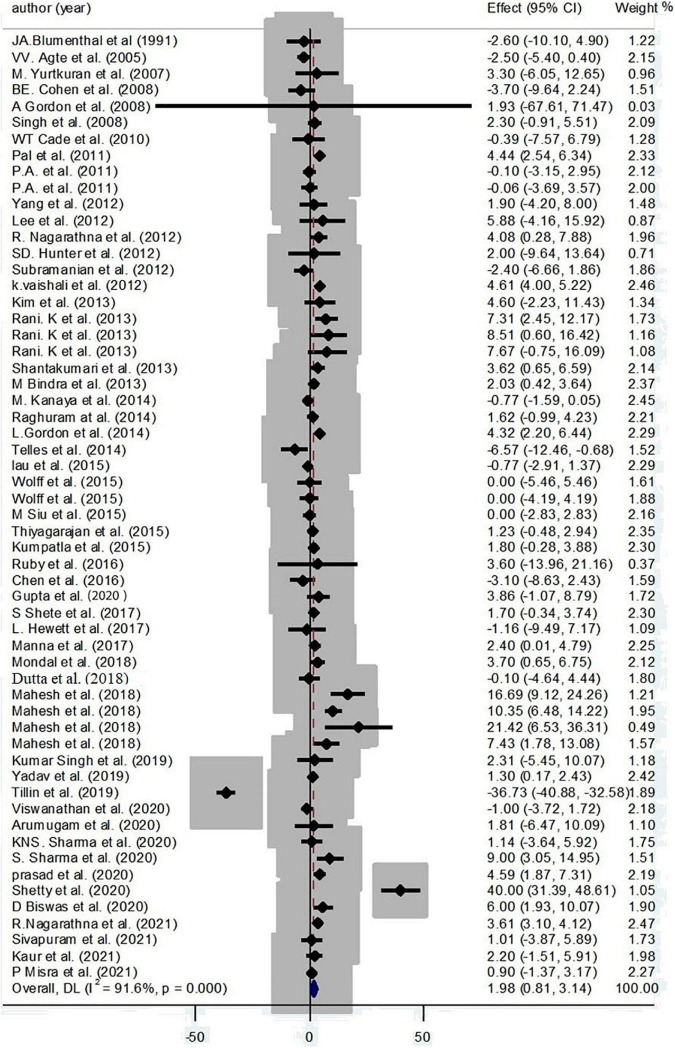
Forest plot for the association between yoga exercise and HDL-C (Random-effect model).

A positive effect of yoga on HDL-C has illustrated by collecting 58 effect sizes (1.98 mg/dl; 95% CI: 0.81, 3.14; *P* < 0.001; *I*^2^ = 91.6%, *P*_heterogeneity_ < 0.001) (Figure 4).

Based on subgroup analysis, continent, condition, and gender were considered as the main sources of heterogeneity. The subgroup analysis based on continent has cleared a positive effect of yoga on HDL-C among Asian participants (3.19 mg/dl; 95% CI: 2.89, 3.48; *P* < 0.001; *I*^2^ = 81.3%, *P*_heterogeneity_ < 0.001). Also, a positive effect of yoga on HDL-C levels was found among patients with type 2 diabetes (3.75 mg/dl; 95% CI: 3.41, 4.10; *P* < 0.001; *I*^2^ = 81.9%; *P*_heterogeneity_ < 0.001) and hypertension (6.18 mg/dl; 95% CI: 4.25, 8.10; *P* < 0.001; *I*^2^ = 79.6%; *P*_heterogeneity_ < 0.001) (Supplementary Table 4).

Based on the result of meta-regression, no effect was found for age on the effect sizes (regression coefficient = 0.03; 95%CI: −0.23, 0.30; *P* = 0.780) (Supplementary Figure 5).

According to the result of Egger’s test and funnel plot of 58 studies, no evidence of publication bias was found (*P* = 0.396) (Supplementary Figure 6). Moreover, the sensitivity analysis indicated that the final estimates did not change by the omission of any included studies.

#### Triglycerides

**FIGURE 5:**
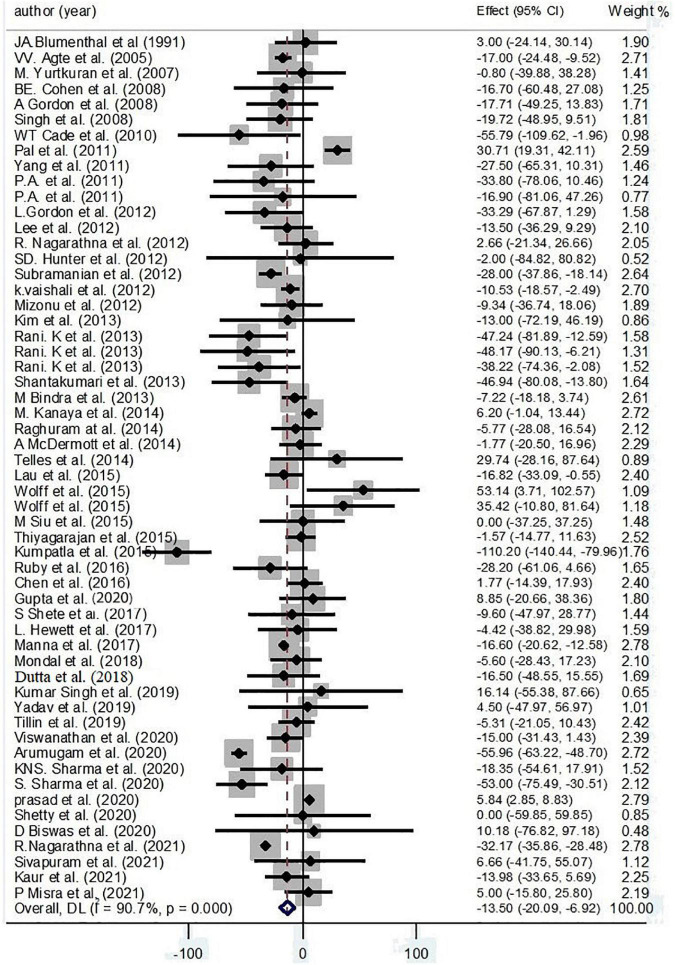
Forest plot for the association between yoga exercise and TG (Random-effect model).

By collecting 58 effect sizes, we found an inverse effect of yoga on TG (−13.50 mg/dl; 95% CI: −20.09, −6.92; *P* < 0.001; *I*^2^ = 90.7%, *P*_heterogeneity_ < 0.001) (Figure 5).

Based on subgroup analysis, continent, condition, and gender were considered the main sources of heterogeneity. The subgroup analysis based on continent has illustrated an inverse effect of yoga on TG among Asian participants (−13.45 mg/dl; 95% CI: −15.08, −11.81; *P* < 0.001; *I*^2^ = 92.3%, *P*_heterogeneity_ < 0.001). Also, an inverse effect of yoga on TG levels was found among healthy practitioners (−15.25 mg/dl; 95% CI: −18.55, −11.95; *P* < 0.001; *I*^2^ = 49.2%, *P*_heterogeneity_ = 0.032) and patients with CKD (−18.03 mg/dl; 95% CI: −38.17, 2.11; *P* = 0.079; *I*^2^ = 0.0%, *P*_heterogeneity_ = 0.471) and type 2 diabetes (−29.96 mg/dl; 95% CI: −32.66, −27.27; *P* < 0.001; *I*^2^ = 87.0%, *P*_heterogeneity_ < 0.001) (Supplementary Table 5).

Based on the result of meta-regression, no effect was found for age on the effect sizes (regression coefficient = 0.68; 95%CI: −0.26, 1.64; *P* = 0.153) (Supplementary Figure 7).

According to the result of Egger’s test and funnel plot of 56 studies, no evidence of publication bias was found (*P* = 0.781) (Supplementary Figure 8). Moreover, the sensitivity analysis of included trials did not affect the final pooled amounts.

#### Very Low-Density Lipoprotein Cholesterol

**FIGURE 6:**
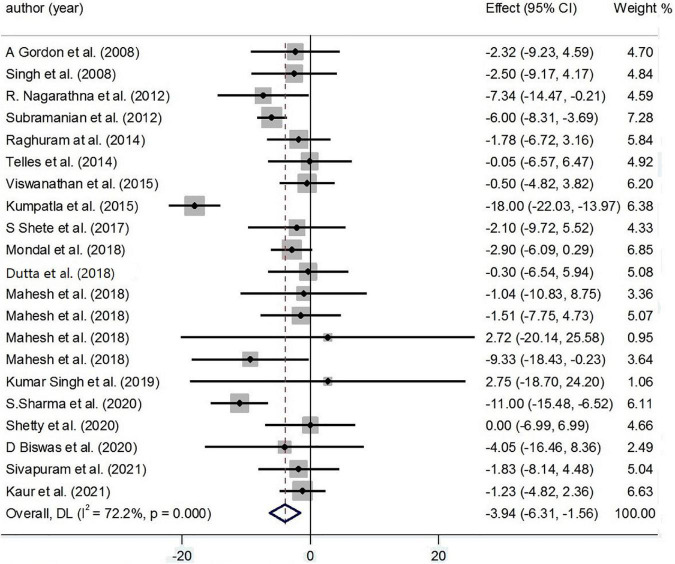
Forest plot for the association between yoga exercise and VLDL-C (Random-effect model).

Findings from 21 effect sizes have detected an inverse effect of yoga on VLDL-C (−3.94 mg/dl; 95%CI: −6.31, −1.56; *P* < 0.001; *I*^2^ = 72.2%, *P*_heterogeneity_ < 0.001) (Figure 6).

Based on subgroup analysis, condition, duration, and gender were considered the main sources of heterogeneity. The subgroup analysis based on condition has shown an inverse effect of yoga on VLDL-C levels among healthy practitioners (−5.67 mg/dl; 95% CI: −7.88, −3.46; *P* < 0.001; *I*^2^ = 0.0%, *P*_heterogeneity_ = 0.337) and patients with type 2 diabetes (−5.81 mg/dl; 95% CI: −7.44, −4.18; *P* < 0.001; *I*^2^ = 87.5%, *P*_heterogeneity_ < 0.001) (Supplementary Table 6).

Based on the result of meta-regression, no effect was found for age on the effect sizes (regression coefficient = 0.20; 95%CI: −0.24, 0.65; *P* = 0.331) (Supplementary Figure 9).

According to the result of Egger’s test and funnel plot of 53 studies, no evidence of publication bias was found (*P* = 0.304) (Supplementary Figure 10). Moreover, the sensitivity analysis of included trials did not affect the final pooled amounts.

## Discussion

In the present meta-analysis, we found out from several articles published in 1991 and since then that yoga had decreased TC, LDL-C, TG, and VLDL-C and increased HDL-C among yoga practitioners.

Along with our findings, some articles have shown improvement in lipid profiles. Many researchers have noted that the practice of yoga and yoga-based programs may have a positive influence on body composition and blood lipid profiles (60, 61), mindfulness may help improve chronic diseases by increasing the individual’s ability to encounter challenges with more efficient coping strategies (62, 63), also it has been reported that modification and stress management educational programs lead to significant improvement in the subjective wellbeing scores and can therefore make an appreciable contribution to primary prevention as well as management of diseases (64). For instance, yoga has decreased TC and LDL-C among Asian athletes through its strong potential as a lifestyle management skill in the Indian population (5). Since yoga stimulates metabolism, it had significant effects on improving TC levels in patients with MetS and type 2 diabetes (5). Not only does practicing yoga regularly improve the flexibility but it also has an effective influence on the function of both endocrine and exocrine glands of the body (64), increased B-cells sensitivity of the pancreas to glucose signals (65), and improved insulin sensitivity as well as a decline in insulin resistance (66), which leads to a reduction in both fasting blood glucose (FBG) and postprandial glucose (PPBG) levels and better glycemic control (64, 67).

It has been shown that HDL-C levels have improved among patients with MetS. The potential mechanism behind this has shown yoga improves several MetS components, suggesting that yoga may prevent or improve atherosclerosis (51). The improvement in lipid profile by practicing yoga could be due to increased hepatic lipase and lipoprotein lipase, which can increase the uptake of TGs by adipose tissue and affect lipoprotein metabolism (7). On the other hand, yoga improves LDL-C receptor sensitivity, receptor-mediated endocytosis, and receptor recycling (3). Also, the regulatory effect of yoga on HDL-C is mediated through a reverse cholesterol transport mechanism that includes macrophage cholesterol efflux in arteries (3). In addition, Indian traditional yoga practices are known as an antioxidant or anti-inflammatory against such diseases by replacing inflammatory markers and metabolic risk factors (68, 69). Overall, the possible reason for the reduction in TG, TC, and LDL-C; as well as elevation in HDL-C is that yoga interventions, especially deep breathing, stretching, and flexibility exercises, increased metabolism and utilization of blood lipids and lipoprotein for energy production (33). For instance, in a study, one participant reported that her medication reduced and improved her clinical status after doing yoga every day (64). Studies presented that TG levels were improved by yoga among patients with CKD and type 2 diabetes (12, 15, 32, 35). Also, by following a regular exercise program, VLDL-C was reduced among type 2 diabetes yoga workers (19, 55). Yoga could also assist in the redistribution of body fat and the reduction of abdominal obesity (7). Also, Thind et al. and Cramer et al. concluded yoga improved lipid status in all participants who had T2DM and MetS (70, 71).

Whereas some studies that accessed the effect of yoga interventions on lipid profiles found contradictory results. Koertge et al. demonstrated that yoga had no improvement effect on HDL-C and TG levels in coronary artery disease (CAD) participants (72). Also, Dutta et al. stated that yoga did not lower HbA1c significantly among people who had done yoga as compared to those who had not done yoga (73). Contrary results are probably due to the lack of high-quality data and also due to the fact that lipids profile in most of the studies were not evaluated as a primary outcome (73). In addition, the different number of participants at the end of the study who completed the intervention and dropout samples could influence the exact results (72).

This is the first study that concentrates on the effect of yoga on lipid profiles with a wide searching process, also performed by PRISMA guidelines (74). Another strength is that we included a large number of well-designed RCTs with relatively large sample sizes and performed subgroup analyses that focused on this effectiveness in different diseases and situations. In most of the studies both gender participated, also RCTs with more than 12 weeks have shown better results on lipid profiles (24). However, some limitations must be considered in the current study, for instance; BMI was not reported in many studies and they had not measured the intensity of yoga, besides most of the RCTs were not blinded and all participants were aware of why they did yoga. Many of the included studies were conducted in India, therefore the results may not be generalizable to all countries. Moreover, we did not study this effect on heart rate, body composition, or sleep quality.

## Conclusion

To conclude, the results of our meta-analysis expand the evidence that yoga had a striking effect on balancing lipid profiles. However, heterogeneity among studies was notable. Further studies are needed to clarify our findings.

## Data Availability Statement

The original contributions presented in this study are included in the article/Supplementary Material, further inquiries can be directed to the corresponding author/s.

## Author Contributions

ED and DG designed the article and wrote syntaxes for primary and advanced searchings from PubMed/Medline, Scopus, Web of Science, and the Cochrane and performed first and second screenings for exclusion and inclusion, eventually, 53 articles were included to our article after the final screening. ED, DG, and VB wrote the body of the article and grammatically checked the possible mistakes for all part of the passages. DG and MD extracted the emergency data from all 53 included articles carefully, evaluated addition quality of the studies based on the Cochrane guideline by the following criteria: random sequence generation, allocation concealment, blinding, incomplete outcome data, selective outcome reporting, and other possible sources of bias. ED performed data analyses using Stata software, version 14 from the data extracted information and tables. All authors contributed to the article and approved the submitted version.

## Conflict of Interest

The authors declare that the research was conducted in the absence of any commercial or financial relationships that could be construed as a potential conflict of interest.

## Publisher’s Note

All claims expressed in this article are solely those of the authors and do not necessarily represent those of their affiliated organizations, or those of the publisher, the editors and the reviewers. Any product that may be evaluated in this article, or claim that may be made by its manufacturer, is not guaranteed or endorsed by the publisher.
