# Supplementary material for: The Effect of Yoga on the Lipid Profile: A Systematic Review and Meta-Analysis of Randomized Clinical Trials
Source: Front Nutr. 2022 Jul 14;9:942702. doi: 10.3389/fnut.2022.942702 (PMC9329825; doi:10.3389/fnut.2022.942702)
Supplement: Supplementary file 1 [file Data_Sheet_1.docx]

| **Study(year)**  **Supplementary Table 1**. Cochrane quality assessment | **Random Sequence Generation** | **Allocation concealment** | **Reporting bias** | **Performance bias** | **Detection bias** | **Attrition bias** | **Other sources of bias** |
| --- | --- | --- | --- | --- | --- | --- | --- |
| Blumenthal et al(1991) (45) | **L** | **U** | **H** | **U** | **U** | **H** | **U** |
| Agte et al.(2005)(43) | **H** | **H** | **H** | **H** | **H** | **U** | **U** |
| Yurtkuran et al(2007)(59) | **L** | **H** | **H** | **H** | **H** | **U** | **U** |
| Cohen et al.(2008)(46) | **L** | **L** | **H** | **H** | **H** | **U** | **L** |
| Gordon et al.(2008)(55) | **L** | **U** | **L** | **U** | **U** | **L** | **U** |
| Singh et al.(2008)(37) | **U** | **H** | **L** | **U** | **U** | **U** | **L** |
| Cade et al.(2010)(54) | **L** | **U** | **H** | **U** | **U** | **L** | **L** |
| Pal et al.(2011)(29) | **L** | **L** | **L** | **L** | **U** | **U** | **L** |
| Yang et al.(2011)(6) | **L** | **H** | **H** | **U** | **H** | **L** | **H** |
| P.A. et al.(2011)(27) | **U** | **L** | **H** | **U** | **U** | **U** | **L** |
| Mizonu et al.(2012)(57) | **U** | **H** | **H** | **U** | **U** | **U** | **U** |
| vaishali et al.(2012)(17) | **L** | **U** | **H** | **U** | **U** | **L** | **U** |
| Subramanian et al.(2012)(39) | **H** | **H** | **L** | **H** | **U** | **L** | **H** |
| Hunter et al.(2012)(47) | **L** | **L** | **H** | **U** | **U** | **U** | **L** |
| Nagarathna et al.(2012)(26) | **L** | **H** | **L** | **H** | **H** | **H** | **L** |
| Lee et al.(2012)(52) | **H** | **H** | **H** | **H** | **H** | **U** | **U** |
| Gordon et al.(2012)(15) | **L** | **L** | **H** | **U** | **U** | **U** | **U** |
| Rani. K et al.(2013)(13) | **U** | **U** | **H** | **U** | **U** | **U** | **L** |
| Bindra et al.(2013)(20) | **U** | **U** | **H** | **U** | **U** | **U** | **U** |
| Kim et al.(2013)(51) | **L** | **H** | **H** | **H** | **H** | **U** | **H** |
| Shantakumari et al.(2013)(7) | **L** | **H** | **H** | **H** | **H** | **U** | **U** |
| Raghuram at al.(2014)(31) | **L** | **H** | **L** | **H** | **H** | **U** | **L** |
| Kanaya et al.(2014)(48) | **L** | **L** | **H** | **U** | **U** | **U** | **L** |
| Telles et al.(2014)(40) | **L** | **L** | **L** | **U** | **U** | **L** | **L** |
| Dermott et al.(2014)(22) | **L** | **H** | **H** | **U** | **U** | **L** | **U** |
| Wolff et al.(2015)(53) | **L** | **L** | **H** | **H** | **H** | **H** | **H** |
| Thiyagarajan et al.(2015)(41) | **L** | **H** | **H** | **U** | **U** | **L** | **U** |
| Kumpatla et al.(2015)(19) | **L** | **H** | **L** | **H** | **U** | **L** | **U** |
| lau et al.(2015)(5) | **H** | **H** | **L** | **H** | **U** | **L** | **L** |
| Siu et al.(2015)(50) | **L** | **H** | **H** | **L** | **U** | **H** | **L** |
| Ruby et al.(2016)(49) | **L** | **L** | **H** | **U** | **U** | **L** | **U** |
| Chen et al.(2016)(2) | **L** | **H** | **H** | **H** | **H** | **L** | **L** |
| Shete et al.(2017)(34) | **L** | **U** | **L** | **U** | **U** | **L** | **L** |
| Hewett et al.(2017)(56) | **L** | **L** | **H** | **L** | **L** | **L** | **L** |
| Manna et al.(2017)(21) | **L** | **H** | **H** | **U** | **U** | **U** | **L** |
| Mondal et al.(2018)(24) | **H** | **H** | **L** | **H** | **U** | **U** | **L** |
| Dutta et al.(2018)(28) | **U** | **U** | **L** | **U** | **U** | **U** | **U** |
| Mahesh et al.(2018)(25) | **L** | **L** | **H** | **U** | **U** | **U** | **U** |
| Kumar Singh et al.(2019)(36) | **H** | **H** | **L** | **U** | **U** | **U** | **L** |
| Tillin et al.(2019)(58) | **H** | **L** | **H** | **H** | **H** | **U** | **L** |
| Yadav et al.(2019)(44) | **L** | **H** | **H** | **H** | **U** | **L** | **U** |
| Viswanathan et al.(2020)(42) | **L** | **L** | **L** | **U** | **U** | **L** | **U** |
| Arumugam et al.(2020)(12) | **U** | **U** | **H** | **U** | **U** | **L** | **L** |
| Sharma et al.(2020)(33) | **L** | **L** | **H** | **U** | **U** | **L** | **U** |
| Sharma et al.(2020)(32) | **L** | **L** | **L** | **U** | **U** | **L** | **U** |
| prasad et al.(2020)(30) | **L** | **L** | **H** | **U** | **U** | **U** | **U** |
| Shetty et al.(2020)(35) | **L** | **U** | **L** | **U** | **U** | **L** | **L** |
| Biswas et al.(2020)(14) | **L** | **H** | **L** | **H** | **H** | **L** | **H** |
| Gupta et al.(2020)(16) | **L** | **L** | **H** | **U** | **U** | **U** | **L** |
| Nagarathna et al.(2021)(3) | **L** | **H** | **L** | **H** | **H** | **L** | **L** |
| Sivapuram et al.(2021)(38) | **L** | **L** | **L** | **U** | **U** | **L** | **U** |
| Kaur et al.(2021)(18) | **L** | **H** | **L** | **H** | **H** | **L** | **H** |
| Misra et al.(2021)(23) | **L** | **U** | **H** | **U** | **U** | **L** | **U** |

L, low risk of bias; H, high risk of bias; U, unclear risk of bias

**Supplementary Table 2.** Subgroup analysis based on fixed-effects models for the effect of yoga exercise on total cholesterol

| **Subgroup** | **Effect sizes**  **(n)** | **Effect sizes**  **(95% CI)** | **I^2^**  **(%)** | ***P***  **Heterogeneity** | ***P***  **Within** | ***P***  **Between** |
| --- | --- | --- | --- | --- | --- | --- |
| **Overall** | 55 | -10.34 (-11.54, -9.14) | 82.5 | < 0.001 | < 0.001 |  |
| **Continent** |  |  |  |  |  |  |
| America | 7 | -13.6 (-21.39, -4.77) | 54.3 | 0.041 | 0.002 | 0.161 |
| Asia | 43 | -10.51 (-11.74, -9.28) | 85.5 | < 0.001 | < 0.001 | 0.161 |
| Australia | 2 | -0.89 ( -14.09, 12.30) | 0.0 | 0.612 | 0.894 | 0.161 |
| Europe | 3 | -3.10 (-11.74, 5.55) | 0.0 | 0.562 | 0.482 | 0.161 |
| **Gender** |  |  |  |  |  |  |
| Both | 42 | -10.84 (-12.08, -9.59) | 85.9 | < 0.001 | < 0.001 | <0.05 |
| Male | 6 | -2.06 ( -7.97, 3.84) | 0.0 | 0.977 | 0.494 | <0.05 |
| Female | 7 | -5.85 (-13.19, 1.50) | 12.1 | 0.338 | 0.119 | <0.05 |
| **Duration** |  |  |  |  |  |  |
| >12 w | 47 | -10.14 (-11.35, -8.92) | 84.1 | < 0.001 | < 0.001 | 0.062 |
| <12 w | 8 | -16.72 (-23.53, -9.92) | 57.1 | < 0.001 | < 0.001 | 0.062 |
| **Condition** |  |  |  |  |  |  |
| Healthy | 11 | -11.61 (-16.13, -9.09) | 60.1 | 0.005 | < 0.001 | < 0.001 |
| Metabolic syndrome | 5 | -14.68 (-19.94, -9.41) | 87.9 | < 0.001 | < 0.001 | < 0.001 |
| CKD | 3 | -13.43 (-19.73, -7.13) | 79.4 | 0.008 | < 0.001 | < 0.001 |
| Type 2 diabetes mellitus | 22 | -15.07 (-16.68, -13.46) | 69.0 | < 0.001 | < 0.001 | < 0.001 |
| HIV+CVD | 1 | -5.03 (-25.75, 15.69) | 0.0 | - |  | < 0.001 |
| MI and CAD | 4 | 3.77 (1.18, 6.35) | 75.3 | 0.007 | 0.004 | < 0.001 |
| hypertension | 8 | -3.45 (-11.87, 498) | 0.0 | 0.694 | 0.423 | < 0.001 |
| Obesity | 1 | -0.39 (-21.51, 20.73) | 0.0 | - | 0.971 | < 0.001 |

**Supplementary Table 3.** Subgroup analysis based on fixed-effects models for the effect of yoga exercise on LDL-C

| **Subgroup** | **Effect sizes**  **(n)** | **Effect sizes**  **(95% CI)** | **I^2^**  **(%)** | ***P***  **Heterogeneity** | ***P***  **Within** | ***P***  **Between** |
| --- | --- | --- | --- | --- | --- | --- |
| **Overall** | 53 | -8.51 (-9.68, -7.34) | 75.0 | < 0.001 | < 0.001 |  |
| **Continent** |  |  |  |  |  |  |
| America | 7 | -3.08 (10.73, 4.58) | 0.0 | 0.476 | 0.431 | 0.010 |
| Asia | 41 | -8.92 (-10.11, -7.72) | 79.1 | < 0.001 | < 0.001 | 0.010 |
| Australia | 2 | 6.11 (-5.6, 17.81) | 0.0 | 0.897 | 0.307 | 0.010 |
| Europe | 3 | 0.23 (-9.79, 10.24) | 0.0 | 0.760 | 0.965 | 0.010 |
| **Gender** |  |  |  |  |  |  |
| Both | 40 | -8.73 (−9.95, -7.51) | 79.6 | < 0.001 | < 0.001 | 0.238 |
| Male | 6 | -8.01 (−13.07, -2.94) | 47.2 | 0.092 | 0.002 | 0.238 |
| Female | 7 | -3.09 (−9.55, 3.37) | 0.0 | 0.566 | 0.348 | 0.238 |
| **Duration** |  |  |  |  |  |  |
| >12 w | 46 | -8.57 (−9.75, -7.38) | 78.2 | < 0.001 | < 0.001 | 0.574 |
| <12 w | 7 | -6.57 (−13.42, 0.28) | 0.0 | 0·968 | 0.060 | 0.574 |
| **Condition** |  |  |  |  |  |  |
| Healthy | 11 | -4.98 (−8.43, -1.53) | 60.1 | 0·005 | 0.005 | < 0.001 |
| Metabolic syndrome | 5 | -2.27 (-7.85, 3.31) | 53.2 | 0.093 | 0.425 | < 0.001 |
| CKD | 2 | -10.49 (−17.49, -3.50) | 0.0 | 0·941 | 0.003 | < 0.001 |
| Type 2 diabete mellitus | 22 | -10.76 (-12.14, -9.38) | 84.4 | < 0.001 | < 0.001 | < 0.001 |
| HIV+CVD | 1 | 6.95 (-10.39, 24.29) | 0.0 | - | 0.432 | < 0.001 |
| MI and CAD | 4 | 3.86 (-1.01, 8.74) | 6.7 | 0.360 | 0.121 | < 0.001 |
| hypertension | 8 | -2.44 (-9.29, 4.40) | 0.0 | 0.774 | 0.484 | < 0.001 |
| Obesity | 1 | -0.95 (-16.79, 14.89) | 0.0 | - | 0.906 | < 0.001 |

| **Subgroup** | **Effect sizes**  **(n)** | **Effect sizes**  **(95% CI)** | **I^2^**  **(%)** | ***P***  **Heterogeneity** | ***P***  **Within** | ***P***  **Between** |
| --- | --- | --- | --- | --- | --- | --- |
| **Overall** | 58 | 2.52 (2.24, 2.79) | 91.6 | < 0.001 | < 0.001 |  |
| **Continent** |  |  |  |  |  |  |
| America | 6 | -0.82 (-1.62, -0.02) | 0.0 | 0.900 | 0.045 | < 0.001 |
| Asia | 47 | 3.19 (2.89, 3.48) | 81.3 | < 0.001 | < 0.001 | < 0.001 |
| Australia | 2 | -0.72 (-6.16, 4.72) | 0.0 | 0.891 | < 0.001 | < 0.001 |
| Europe | 3 | -14.35 (-16.94,-11.76) | 98.9 | < 0.001 | < 0.001 | < 0.001 |
| **Gender** |  |  |  |  |  |  |
| Both | 45 | 2.52 ( 2.24, 2.81) | 93.4 | < 0.001 | < 0.001 | 0.727 |
| Male | 5 | 2.66 ( 1.48, 3.84) | 2.3 | 0.394 | < 0.001 | 0.727 |
| Female | 8 | 1.79 ( −0.10, 3.67) | 0.0 | 0.432 | 0.063 | 0.727 |
| **Duration** |  |  |  |  |  |  |
| >12 w | 50 | 2.51 (2.24, 2.79) | 91.6 | < 0.001 | < 0.001 | 0.999 |
| <12 w | 8 | 2.52 (1.09, 3.95) | 92.9 | < 0.001 | < 0.001 | 0.999 |
| **Condition** |  |  |  |  |  |  |
| Healthy | 11 | -1.15 (-2.18, -0.13) | 96.8 | < 0.001 | 0.027 | < 0.001 |
| Metabolic syndrome | 10 | -0.14 (−0.72, 0.43) | 55.2 | 0.017 | 0.628 | < 0.001 |
| CKD | 3 | 0.97 (−2.43, 4.36) | 0.0 | 0.763 | 0.577 | < 0.001 |
| Type 2 diabetes mellitus | 21 | 3.75 (3.41, 4.10) | 81.9 | < 0.001 | < 0.001 | < 0.001 |
| HIV+CVD | 1 | -0.39 (-7.57, 6.79) | 0.0 | - | 0.915 | < 0.001 |
| MI and CAD | 4 | 3.54 ( 2.26, 4.83) | 33.1 | 0.214 | < 0.001 | < 0.001 |
| hypertension | 7 | 6.18 ( 4.25, 8.10) | 79.6 | < 0.001 | < 0.001 | < 0.001 |
| Obesity | 1 | -6.57 (-12.46, -.0.68) | 100.0 | - | < 0.001 | < 0.001 |

**Supplementary Table 4.** Subgroup analysis based on fixed-effects models for the effect of yoga exercise on HDL-C

| **Subgroup** | **Effect sizes**  **(n)** | **Effect sizes**  **(95% CI)** | **I^2^**  **(%)** | ***P***  **Heterogeneity** | ***P***  **Within** | ***P***  **Between** |
| --- | --- | --- | --- | --- | --- | --- |
| **Overall** | 56 | -12.34 (-13.91, -10.77) | 90.7 | < 0.001 | < 0.001 |  |
| **Continent** |  |  |  |  |  |  |
| America | 8 | 1.51 (-4.81, 7.82) | 28.0 | 0.205 | 0.639 | < 0.001 |
| Asia | 43 | -13.45 (-15.08, -11.81) | 92.3 | < 0.001 | < 0.001 | < 0.001 |
| Australia | 2 | -19.32 (-48.30, 9.67) | 59.7 | 0.115 | 0.192 | < 0.001 |
| Europe | 3 | 3.44 (-10.83, 17.70) | 71.1 | 0.032 | 0.637 | < 0.001 |
| **Gender** |  |  |  |  |  |  |
| Both | 42 | -12.75 (-14.36, -11.14) | 92.8 | < 0.001 | < 0.001 | < 0.001 |
| Male | 6 | -6.86 ( -16.87, 3.16) | 0.0 | 0.692 | 0.180 | 0.180 |
| Female | 8 | -1.84 ( −11.86, 8.18) | 38.6 | 0·122 | 0.719 | 0.719 |
| **Duration** |  |  |  |  |  |  |
| >12 w | 48 | -12.17 ( -13.78, -10.56) | 91.8 | < 0.001 | < 0.001 | < 0.001 |
| <12 w | 8 | -15.58 ( -22.67, -8.49) | 55.9 | 0·026 | < 0.001 | < 0.001 |
| **Condition** |  |  |  |  |  |  |
| Healthy | 11 | -15.25 ( −18.55, -11.95) | 49.2 | 0.032 | < 0.001 | < 0.001 |
| Metabolic syndrome | 10 | -6.38 ( −10.93, -1.82) | 61.6 | 0.005 | 0.006 | 0.006 |
| CKD | 3 | -18.03 ( −38.17, 2.11) | 0.0 | 0.471 | 0.079 | 0.079 |
| Type 2 diabete mellitus | 22 | -29.96 ( -32.66, -27.27) | 87.0 | < 0.001 | < 0.001 | < 0.001 |
| HIV+CVD | 1 | -55.79 (-109.62, -1.98) | 0.0 | - | 0.042 | 0.042 |
| MI and CAD | 4 | 7.07 (4.20, 9.93) | 85.2 | < 0.001 | < 0.001 | < 0.001 |
| Hypertension | 4 | 11.63 (-9.04, 32.30) | 49.8 | 0.113 | 0.270 | 0.270 |
| Obesity | 1 | 29.74 (-28.16, 87.64) | 0.0 | - | 0.314 | 0.314 |

**Supplementary Table 5.** Subgroup analysis based on fixed-effects models for the effect of yoga exercise on TG

| **Subgroup** | **Effect sizes**  **(n)** | **Effect sizes**  **(95% CI)** | **I^2^**  **(%)** | ***P***  **Heterogeneity** | ***P***  **Within** | ***P***  **Between** |
| --- | --- | --- | --- | --- | --- | --- |
| **Overall** | 21 | -4.70 (-5.82, -3.59) | 72.2 | < 0.001 | < 0.001 |  |
| **Gender** |  |  |  |  |  |  |
| Both | 16 | -5.81 (-7.14, -4.48) | 75.9 | < 0.001 | < 0.001 | 0.009 |
| Male | 3 | -1.51 (-4.22, 1.21) | 0.0 | 0.971 | 0.277 | 0.009 |
| Female | 2 | -2.78 (−5.93, 0.83) | 0.0 | 0.610 | 0.084 | 0.009 |
| **Duration** |  |  |  |  |  |  |
| >12 w | 17 | -4.73 (-6.08, -3.38) | 76.0 | < 0.001 | < 0.001 | 0.955 |
| <12 w | 4 | -4.66 (-6.64, -2.67) | 43.7 | 0.149 | < 0.001 | 0.955 |
| **Condition** |  |  |  |  |  |  |
| Healthy | 2 | -5.67 (−7.88, -3.46) | 0.0 | 0.337 | < 0.001 | 0.49 |
| Metabolic syndrome or obesity | 10 | -1.68 (−4.86, 1.50) | 3.7 | 0.005 | 0.299 | 0.49 |
| Type 2 diabete mellitus | 8 | -5.81 (−7.44, -4.18) | 87.5 | < 0.001 | < 0.001 | 0.49 |
| Hypertension | 5 | -3.24 (-7.45, 0.97) | 0.0 | 0.647 | 0.131 | 0.49 |
| Other | 2 | -1.21 (-5.08, 2.66) | 0.0 | 0.715 | 0.540 | 0.49 |

**Supplementary Table 6.** Subgroup analysis based on fixed-effects models for the effect of yoga exercise on VLDL-C
