# Supplementary material for: The Effect of Yoga on the Lipid Profile: A Systematic Review and Meta-Analysis of Randomized Clinical Trials
Source: Front Nutr. 2022 Jul 14;9:942702. doi: 10.3389/fnut.2022.942702 (PMC9329825; doi:10.3389/fnut.2022.942702)
Supplement: Supplementary file 2 [file Data_Sheet_2.docx]

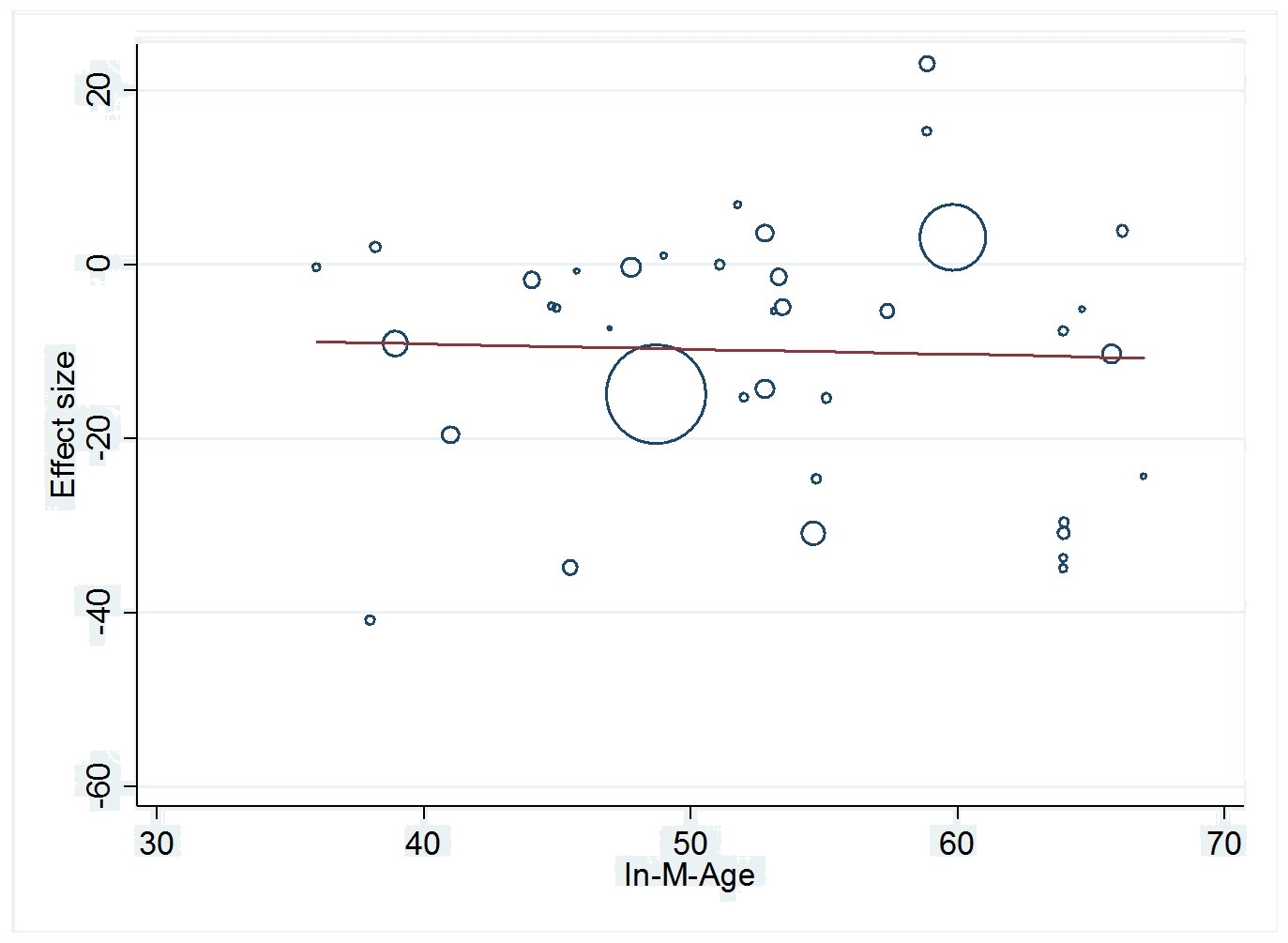


**Supplementary Fig. 1** Meta regression for the association between yoga exercise and TC


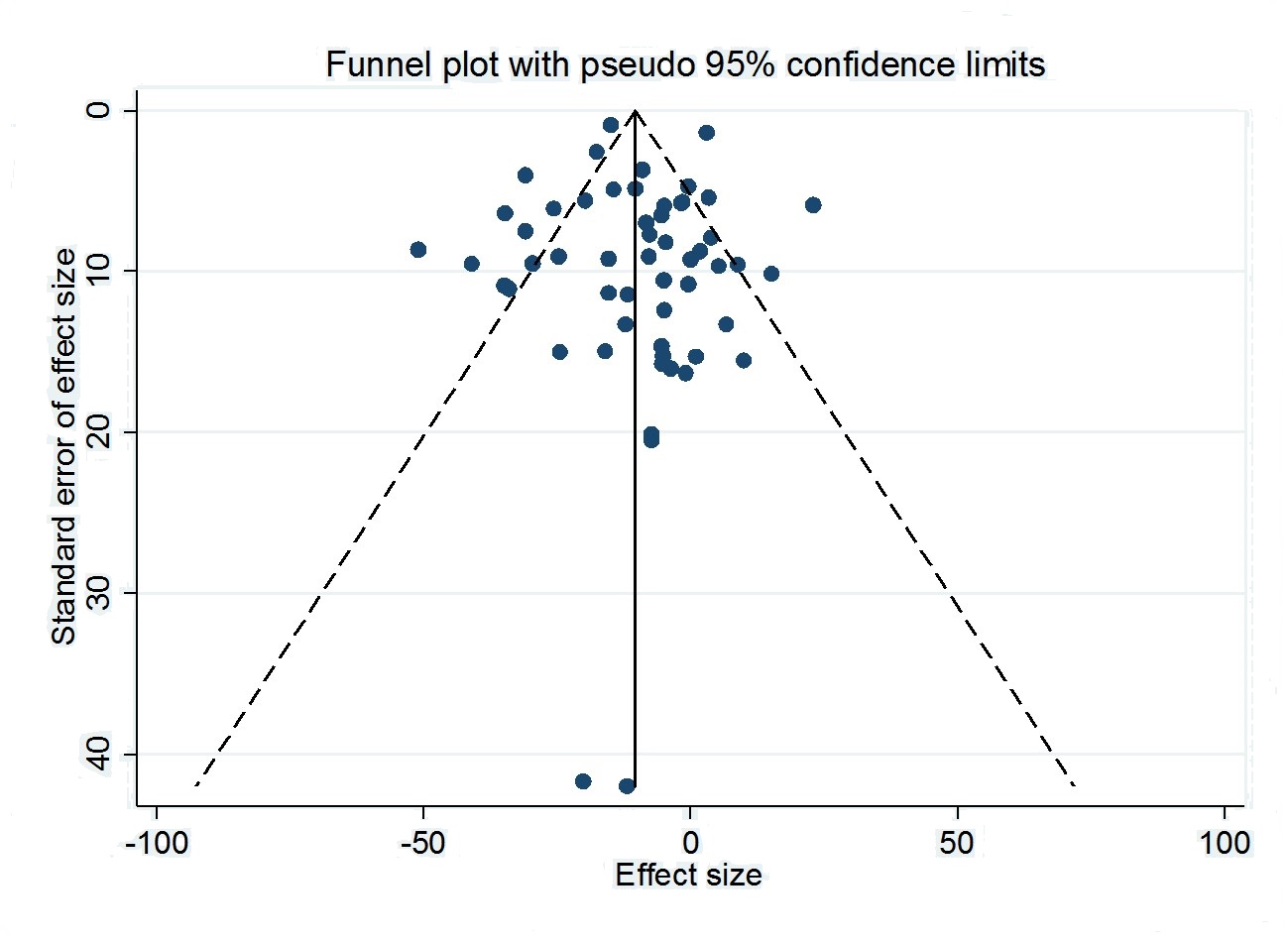


**Supplementary Fig. 2** Funnel plot for the association between yoga exercise and TC


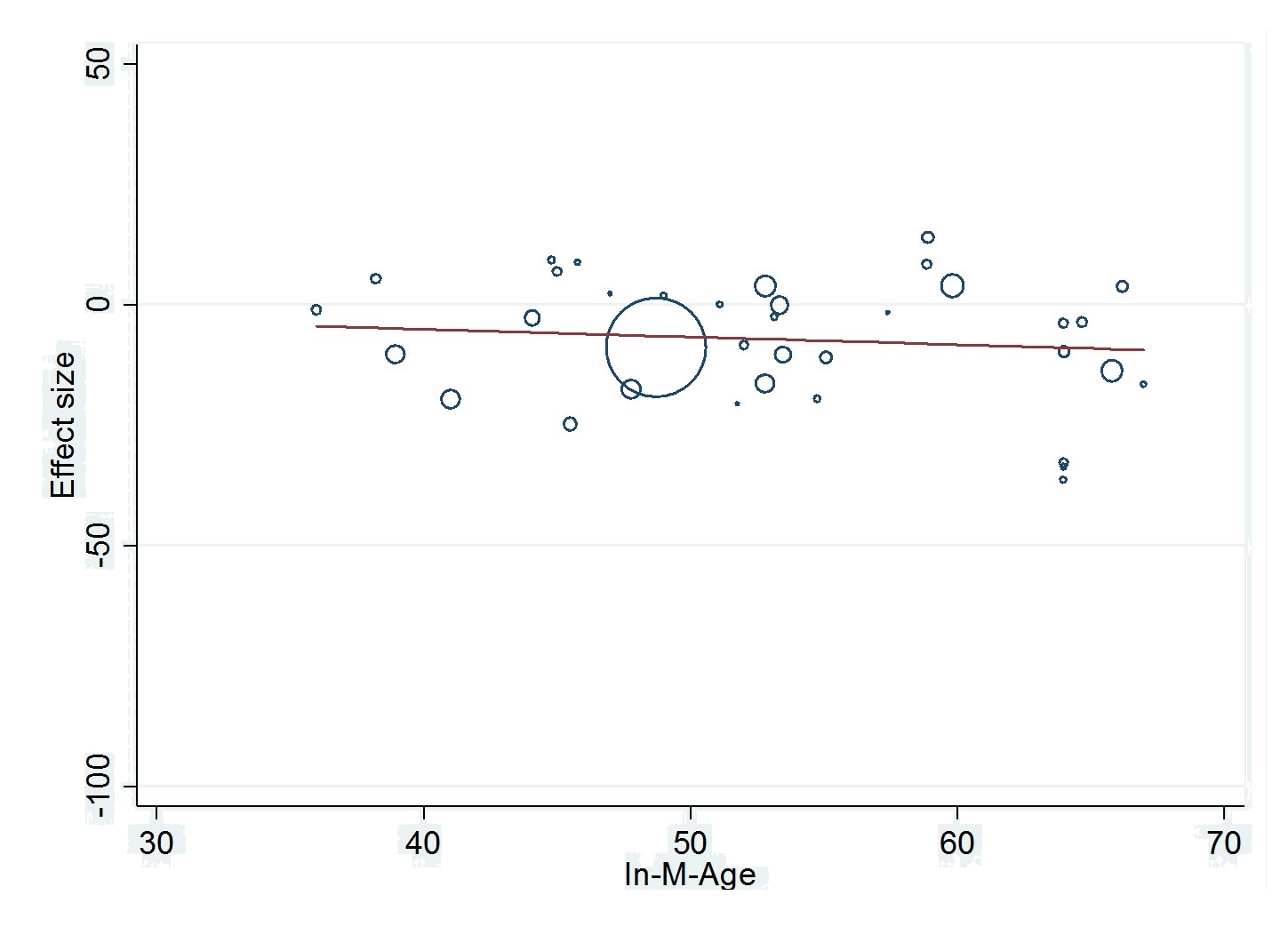


**Supplementary Fig. 3** Meta regression for the association between yoga exercise and LDL-C


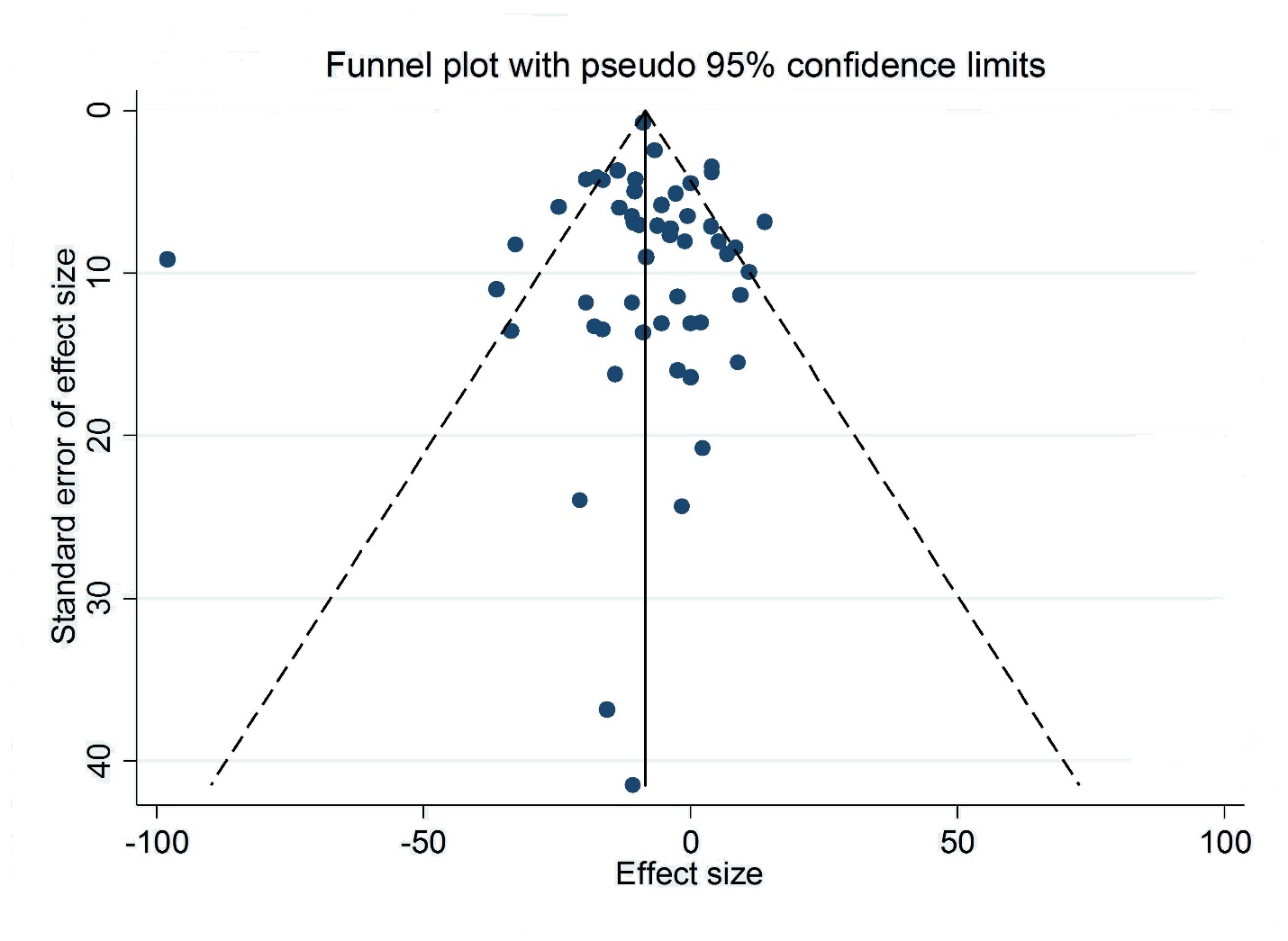


**Supplementary Fig. 4** Funnel plot for the association between yoga exercise and LDL-C


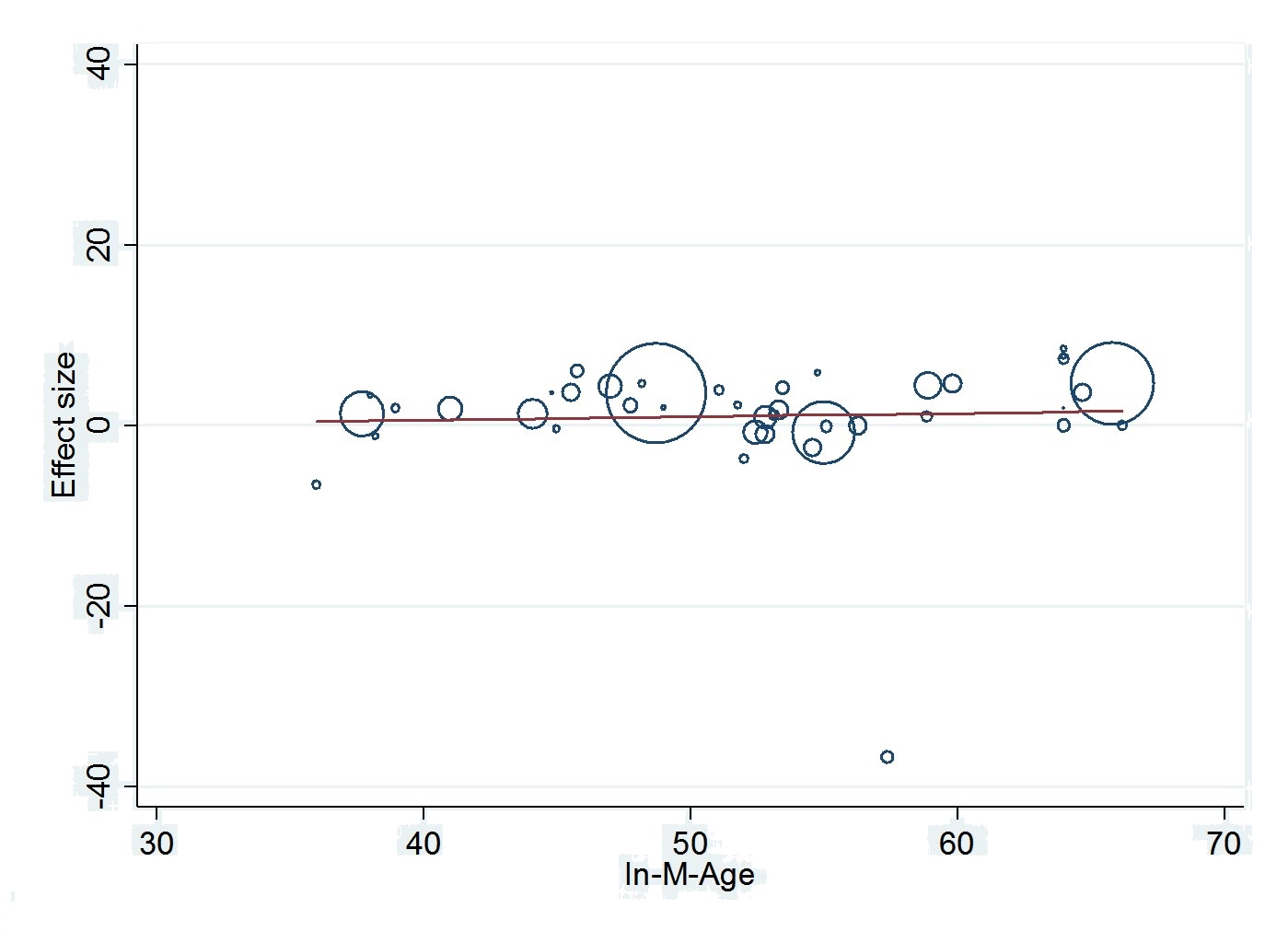


**Supplementary Fig. 5** Meta regression for the association between yoga exercise and HDL-C

C


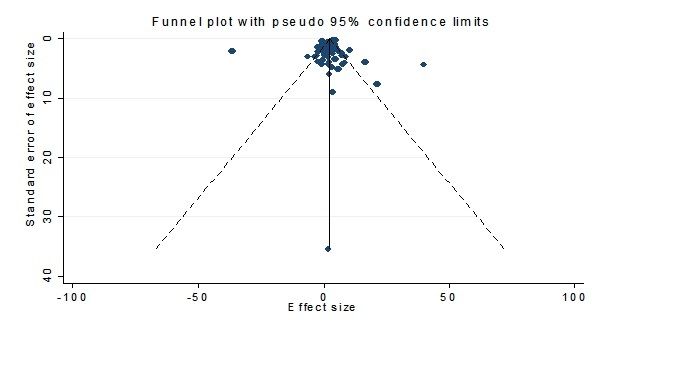


**Supplementary Fig. 6** Funnel plot for the association between yoga exercise and HDL-C


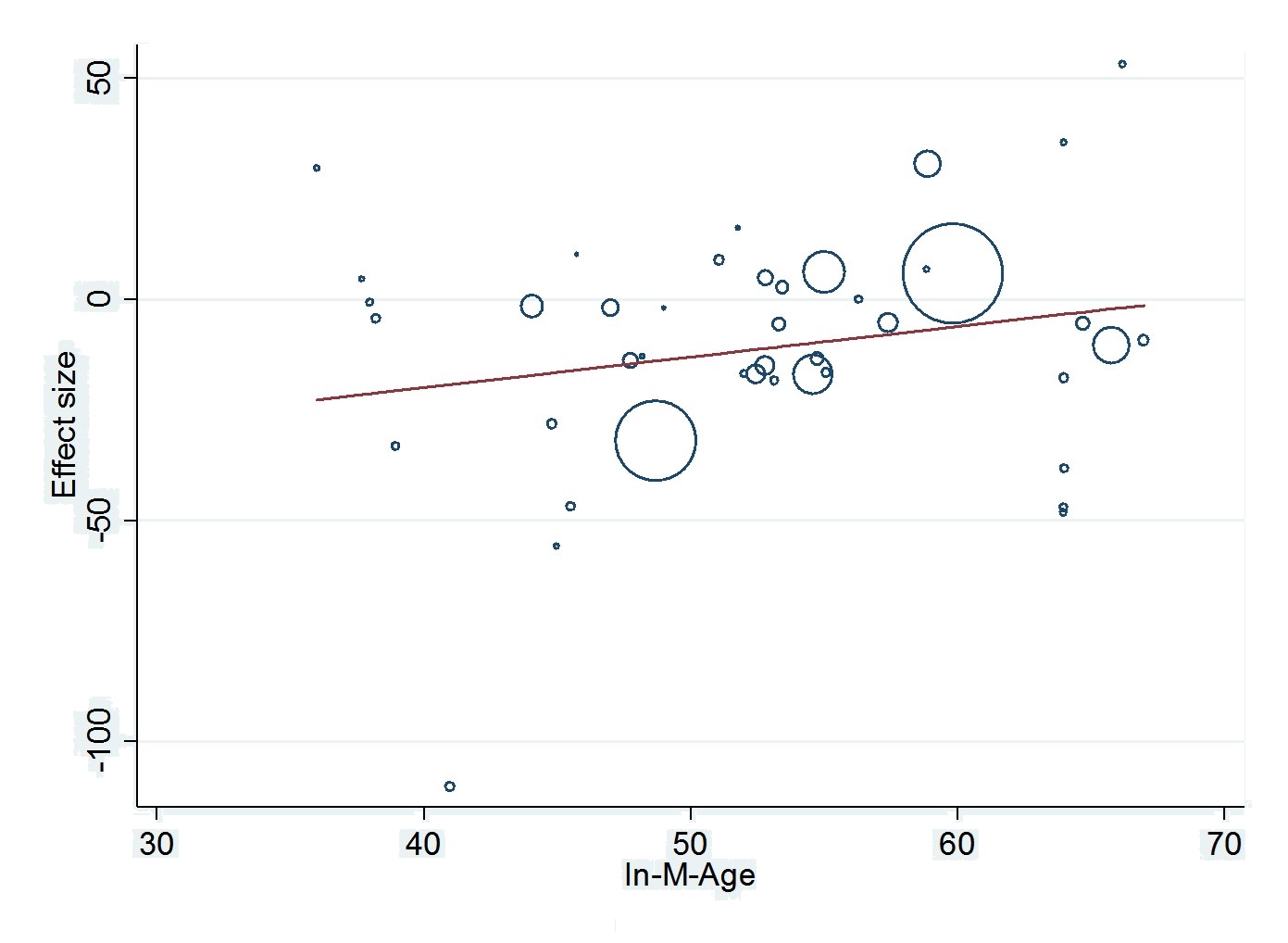


**Supplementary Fig. 7** Meta regression for the association between yoga exercise and TG


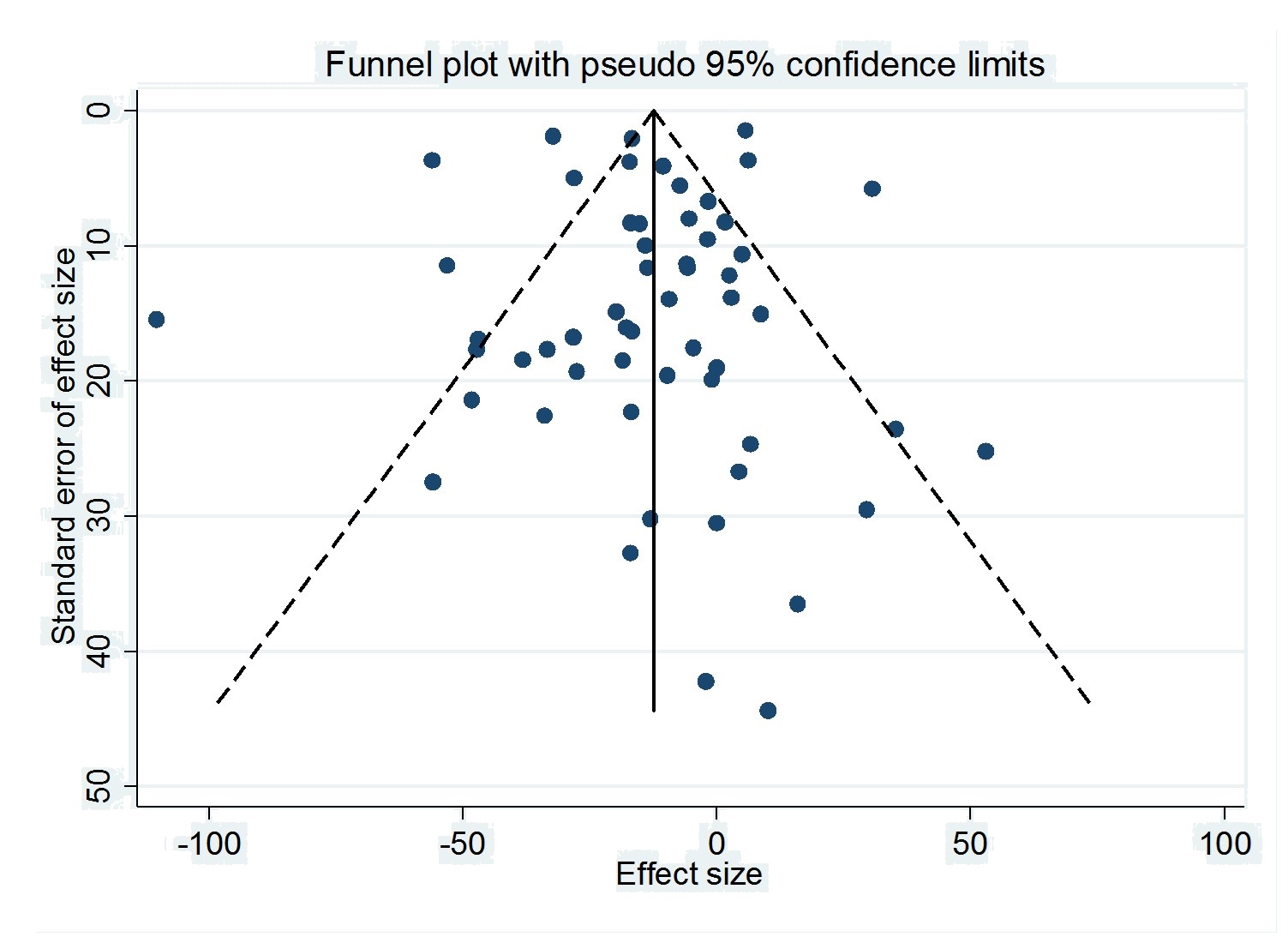


**Supplementary Fig. 8** Funnel plot for the association between yoga exercise and TG


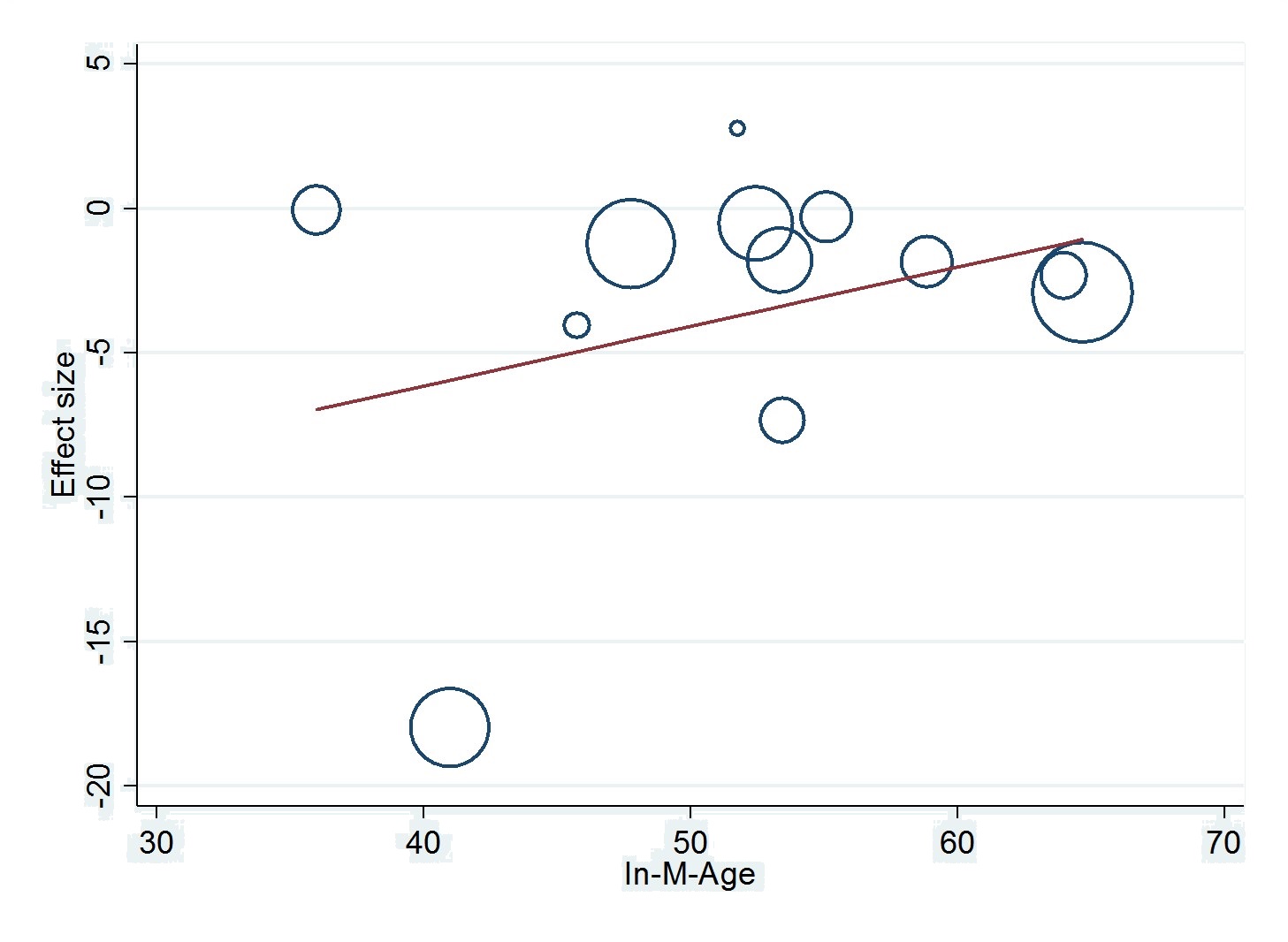


**Supplementary Fig. 9** Meta regression for the association between yoga exercise and VLDL-C


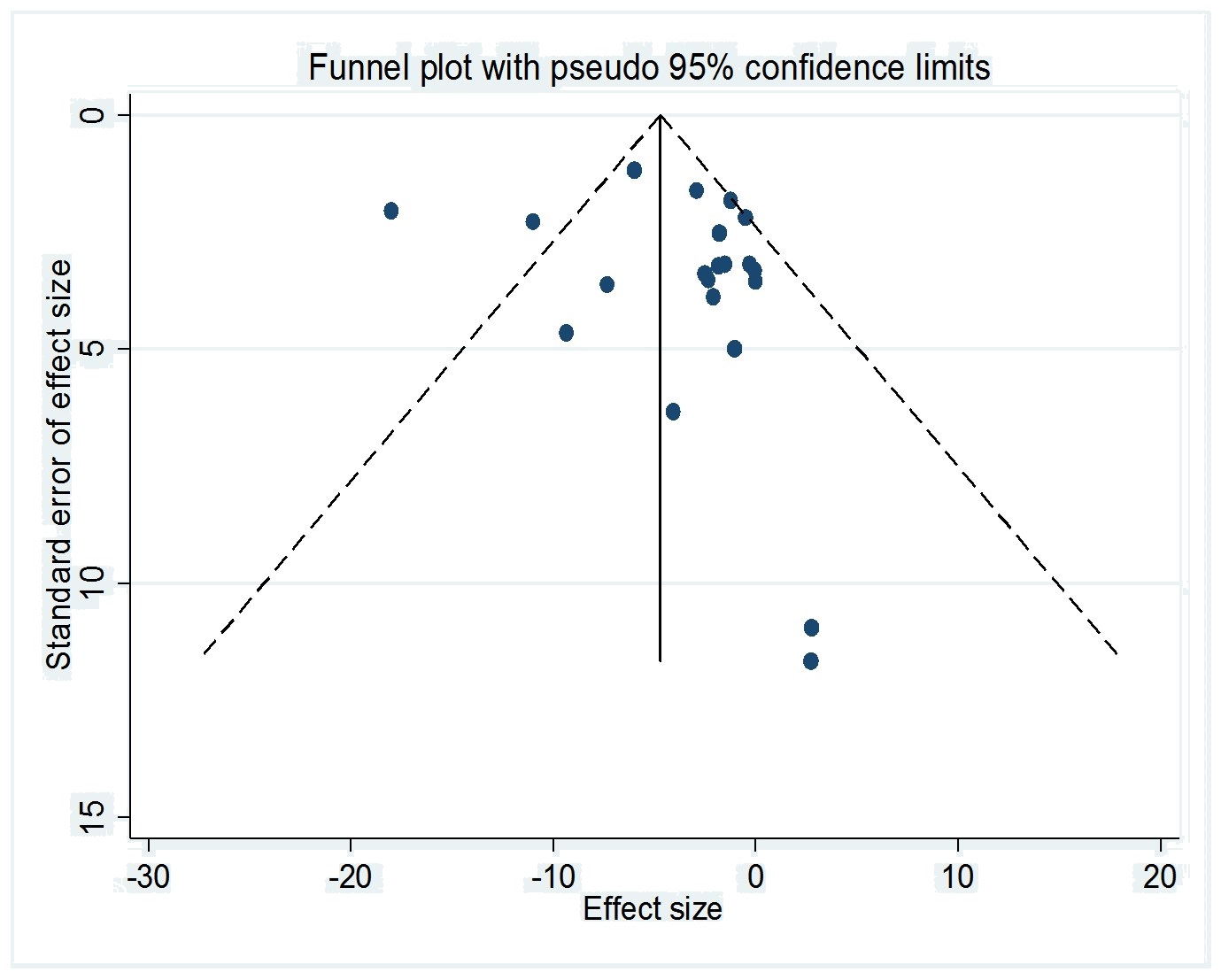


**Supplementary Fig. 10** Funnel plot for the association between yoga exercise and VLDL-C
